# On resolving ambiguities in microbial community analysis of partial nitritation anammox reactors

**DOI:** 10.1038/s41598-019-42882-8

**Published:** 2019-05-06

**Authors:** Laura Orschler, Shelesh Agrawal, Susanne Lackner

**Affiliations:** Technische Universität Darmstadt, Institute IWAR, Chair of Wastewater Engineering, Franziska-Braun-Straße 7, 64287 Darmstadt, Germany

**Keywords:** Applied microbiology, Water microbiology

## Abstract

PCR-based methods have caused a surge for integration of eco-physiological approaches into research on partial nitritation anammox (PNA). However, a lack of rigorous standards for molecular analyses resulted in widespread data misinterpretation and consequently lack of consensus. Data consistency and accuracy strongly depend on the primer selection and data interpretation. An *in-silico* evaluation of 16S rRNA gene eubacterial primers used in PNA studies from the last ten years unraveled the difficulty of comparing ecological data from different studies due to a variation in the coverage of these primers. Our 16S amplicon sequencing approach, which includes parallel sequencing of six 16S rRNA hypervariable regions, showed that there is no perfect hypervariable region for PNA microbial communities. Using qPCR analysis, we emphasize the significance of primer choice for quantification and caution with data interpretation. We also provide a framework for PCR based analyses that will improve and assist to objectively interpret and compare such results.

## Introduction

Partial nitritation anammox (PNA), a significant breakthrough as an energy- and cost-saving alternative to conventional biological nitrogen removal^1–3^, demands a fine balance of operational conditions that support the characteristic microbial composition of ammonium oxidizing bacteria (AOB) and anaerobic ammonium-oxidizing bacteria (AnAOB). Researchers are adopting the combination of microbial ecology and physiology, also known as eco-physiological approach^4–9^, to gain a more fundamental understanding and to optimize PNA processes.

Modern molecular tools have revolutionized the integration of microbial ecology studies into research on PNA systems by circumventing the limitations of cultivation-based approaches^10^. The use of high-throughput 16S rRNA amplicon sequencing in PNA systems also revealed a microbial composition reaching far beyond AOB and AnAOB^9,11^. In PNA studies, 16S amplicon sequencing is performed on the one hand for microbial community characterization and on the other hand - based on the relative abundance of reads - for quantification. Although recently developed ultrahigh-throughput sequencing technologies now overshadow quantitative polymerase chain reaction (qPCR) method, the ability of qPCR to target microorganisms down to strain level with particular taxonomic or functional markers and the ability for accurate enumeration is indispensable^9^. Therefore, qPCR is used in parallel to validate the quantification results of 16S amplicon sequencing^12,13^.

For engineering purposes, the quantification of the desired microorganisms is often more relevant than the inventory of species present in the reactor, and therefore, qPCR is an invaluable method in the molecular microbial ecologist′s toolbox^14^. Moreover, the interpretation of qPCR results with subsequent translation into reactor performance is the most critical point, because these results support the evaluation of a reactor system. We introduce three PNA studies with similar objectives (application of PNA in the main wastewater treatment line) as examples for comparison, to explain how diverse results are interpreted and translated. These studies compared ecological data and reactor performance to understand which reactor operation strategy might be best applicable for mainstream PNA. Hu *et al*.^15^ investigated a lab-scale sequencing batch reactor (SBR) system and interpreted the reactor turnover based on qPCR results. For AnAOB quantification the primer pair hzsA526F/hzsA1829R was used instead of the previously recommended primer pair hzsA1597F/hzsA1857R^16^. Persson *et al*.^7^ quantified microorganisms in a pilot-scale moving bed biofilm reactor (MBBR) with qPCR and stated a high percentage of anammox by normalizing it with the total bacterial abundance captured using primer 1055f-1392r (V7-V8 hypervariable region). This study also compared AnAOB abundance with Hu *et al*.^15^, even though the primers differed – 16S rRNA gene and hzsA (hydrazine synthase) in latter. Gilbert *et al*.^17^ quantified target microbial members using qPCR and compared the results with Hu *et al*.^15^ and Persson *et al*.^7^, even though other primers were used.

Comparing reactor studies with each other is already challenging due to inherent ecological variability. Additionally, biases pervade PCR based analyses. Therefore, in PCR based methods (like qPCR and 16S rRNA amplicon sequencing) primer selection is the most critical step as also reported in several studies^18–20^. Using primers with wide coverage can lead to overrepresentation, whereas primers with high specificity can lead to underrepresentation^19,21,22^. Thus, PCR based analysis needs a framework, where methods and parameters are kept same to compare different studies, similar to the analytical chemistry framework for wastewater treatment plants (WWTP)^23^ (for example chemical oxygen demand, total suspended solids, pH analyses).

By now, there are some guidelines available for PCR based methods, known as MIQE (minimum information for publication of quantitative real-time PCR experiments), guidelines, which emphasize on better transparency in reporting of experimental data^24,25^. These guidelines help to deal with some critical aspects in research fields such as medicine; food processing; and environmental studies, with respect to the reliability of PCR based methods^26^; false positive signals^27^; reproducibility; and lack of comparability^28–30^. However, MIQE guidelines do not include information about experimental protocols, the influence of primer choice and subsequent data interpretation^26^. In the research field of wastewater treatment, experienced users, therefore, developed standardized step-wise protocols for PCR based methods (such as qPCR and 16S amplicon sequencing), primarily focused on wastewater treatment microbial ecology, addressed to non-specialists to shed light on the dark side of the PCR based experiments^31^. For non-specialists, these protocols are useful, however, detailed information about the impact of primer choice, and microbial community matrices on the data and interpretation of that data in PNA studies, which present their own hurdles, is still missing.

We, therefore, systematically provide insight into how to deal with two major questions: (1) What if selected primers do not tell us everything about the PNA microbial community? (2) Can we compare one PNA system with another based on ecological analysis, even when we select different primers for the same query? We assessed previous PNA literature to determine the commonly used primers and approaches for the interpretation of the results with the link to reactor operation. The impact of primers targeting different hypervariable regions of the 16S rRNA gene was investigated by simultaneously sequencing six of the hypervariable regions of the 16S rRNA gene.

The significance of choosing the right data interpretation approach was evaluated by testing different approaches found in previous literature. Further, we developed a decision tree framework for the standardization of PCR-based analysis for PNA systems.

## Results

### Coverage assessment of known 16S rRNA gene universal primers

We retrieved details about the primers from previous studies, which performed microbial abundance quantification in PNA reactors, to determine the most frequently used primers. We found eight different universal primer pairs, targeting different hypervariable regions of the 16S rRNA gene, which were used in the evaluated studies (Table 1). The most frequently used EUB primer pair targets the hypervariable region V7-V8, i.e., primer pair 1055f-1392r (12 hits). The second and third most commonly used primer pairs belong to the hypervariable region V3-V4, primer pair 338f-518r (6 hits) and primer pair 341f-543r (4 hits), respectively. Moreover, there is huge variability in PCR product size, ranging from product sizes of 123 bp (1396F-1492R) as shortest, to 566 bp (341F-907R) as longest. The size of the PCR product also influences the qPCR results^32^. This assessment revealed that a diverse set of primers had been used to quantify the microbial composition in PNA systems which raises the question whether the selection of the primer pair affects quantification and comparability?

**Table 1:** List of 16S rRNA gene primer pairs that were used in the evaluated studies (based on the literature assessment), with the respective hypervariable (HVR) regions and length in base pairs (bp).

Primer pair	HVR- region	length [bp]	HITS
338f-518r	V3-V4	180	6
341f-543r	V3-V4	202	4
341f-907r	V3-V5	566	2
519f-907r	V4-V5	391	2
515f-806r	V4-V5	291	1
907f-1110r	V6-V7	203	1
1055f-1392r	V7-V8	337	12
1369f-1492r	V8-V9	123	3

Hits refer to the frequency of the respective primer pair found in the evaluated studies, no hits as well as more than one hit is possible.

**Figure 1:**
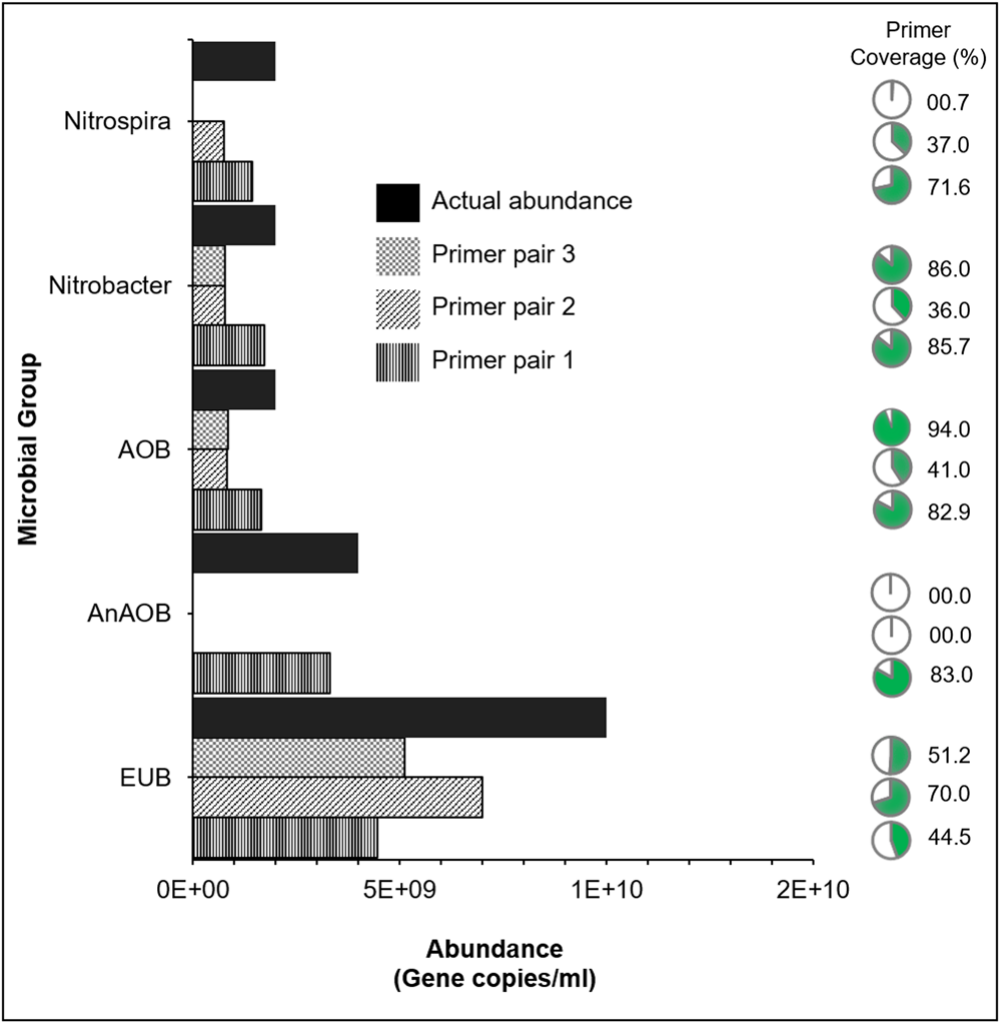
Comparison of the three most widely used EUB primer pairs based on the literature assessment, primer pair 1 (1055f-1392r), primer pair 2 (338f-518r) and primer pair 3 (341f-543r) for the primer coverage and abundance of the microbial groups most relevant for the PNA process (abundance is defined as theoretically calculated value). AnAOB (anaerobic ammonium oxidizing bacteria), (AOB) ammonium oxidizing bacteria, Nitrobacter and Nitrospira (nitrite oxidizing bacteria) and EUB (total eubacteria).

To answer this question, the three most frequently used eubacterial primer pairs in all evaluated studies (primer pair 1:1055f-1392r; primer pair 2: 338f-518r; primer pair 3: 341f-543r) were selected from Table 1 for the *in-silico* PCR analysis (Fig. 1). Based on the current 16S rRNA gene sequence database SILVA (silva132) we studied the total coverage of every primer pair, indicating how much information the respective primer pair provides of the known total eubacterial diversity.

Starting with primer pair 1, the database evaluation showed a total coverage of 44.5% for the eubacterial population. Additionally, the coverage for AOB was about 82.9%, for AnAOB 83.0%, the NOB coverage was 85.7% for *Nitrobacter* and 71.6% for *Nitrospira*. Primer pair 2 had a total coverage of 70.0%, with 41.0% for AOB, no coverage for AnAOB, 36.0% for *Nitrobacter* and 37.0% for *Nitrospira*. Primer pair 3 had a 51.2% coverage for total EUB, 94.0% for AOB, no coverage for AnAOB, 86.0% for *Nitrobacter* and 0.7% *Nitrospira*. These results prove that the qPCR data differs between various PNA studies using different primer pairs.

Previous studies have primarily highlighted that primer selection has a different influence on taxonomic assignments at different taxonomic levels^22,33^. However, here we try to emphasize that primer selection also influences abundance quantification using a theoretical example. Let us consider a hypothetical biomass composition which contains 1.00E + 10 16S rRNA gene copies/mL associated with the eubacterial population, 4.00E + 09 16S rRNA gene copies/mL associated with AnAOB, and 2.00E + 09 16S rRNA gene copies/mL associated with AOB, *Nitrobacter* and *Nitrospira*, respectively. Using the different EUB primers resulted in significant, different theoretical abundances (p-value < 0.01, two-way analysis of variance (ANOVA) analysis) (Fig. 1). Based on the current SILVA database, the total eubacterial population is under-represented using either of the three most commonly used primer pairs. It is even more critical for specific microbial groups in PNA systems, as lack of appropriate primers can lead to false negative results, for example, primer pair 2 does not cover AnAOB and primer pair 3 does not cover AnAOB and *Nitrospira* (similar outcome in multiple sequence alignment, Supplementary Figure 2).

### Coverage of primers that are microbial group-specific

**Figure 2:**
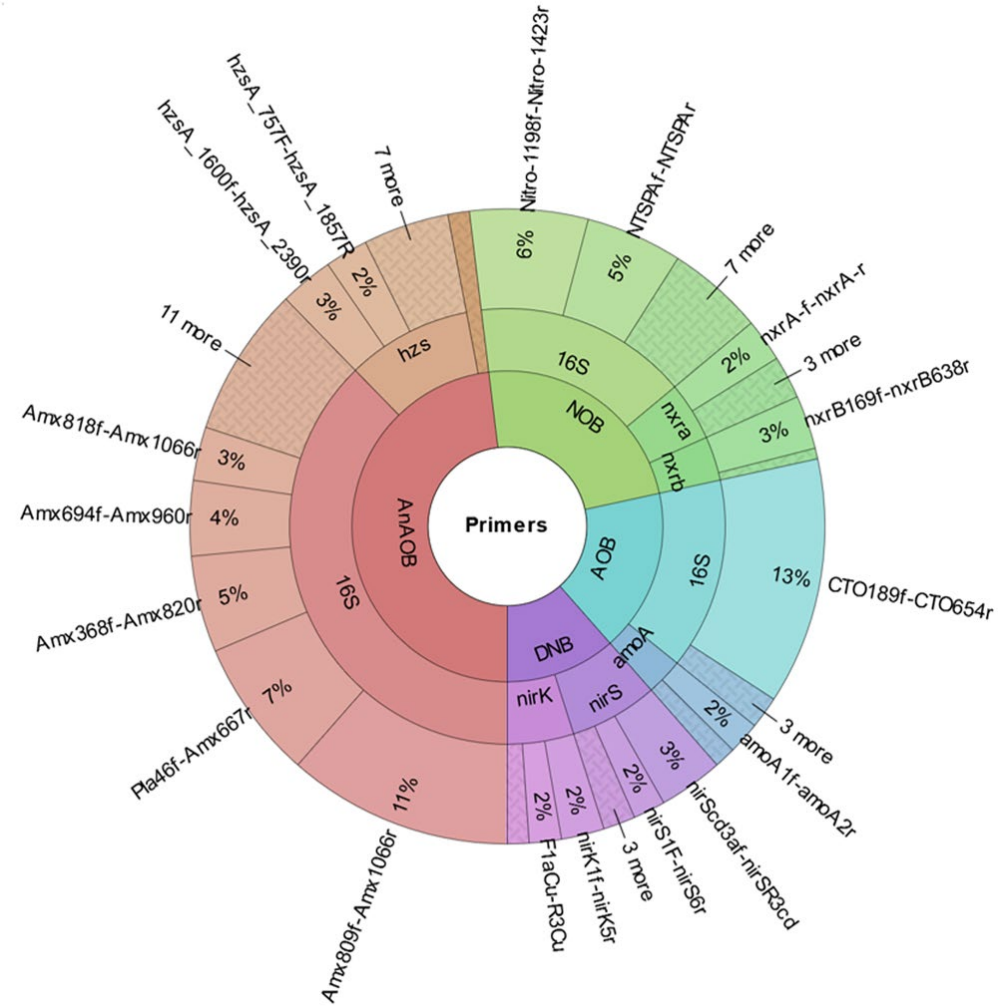
Distribution of the diversity of primer pairs based on the literature survey using certain keywords (i.e., “qPCR”, “anammox”, “wastewater”), for different microbial groups and the percentage of usage. (1) AnAOB: Anaerobic ammonium oxidizing bacteria; (2) AOB: ammonium oxidizing bacteria; (3) NOB: nitrite oxidizing bacteria; and (4) DNB: denitrifying bacteria. (1) 16S: 16S rRNA gene; (2) amoA: ammonia monooxygenase; (3) hzs: hydrazine synthase; (4) nirK: copper-containing nitrite reductase; (5) nirS: cytochrome cd1 type nitrite reductase; (6) nxra: nitrite oxidoreductase, alpha subunit; and (7) nxrb: nitrite oxidoreductase, beta subunit.

The challenge to compare qPCR results from different PNA studies is not just limited to the EUB universal primers. It extends to microbial group-specific primers, too. Similar to EUB primers, a wide range of group-specific primers are used in PNA studies, hampering the comparison of PNA studies that used different group-specific primers. We, therefore, looked at the distribution of different primer pairs that were used in the evaluated studies, and obtained 213 hits for different group-specific primers (including 16S rRNA and functional genes) from the 70 studies (Fig. 2). This survey resulted in the following diversity in primer usage: AOB < DNB (denitrifying bacteria) < NOB < AnAOB. The most commonly used primer pair for AOB was amoA1f-amoA2r; for Anammox it was Amx809f-Amx1066r; Nitro1198f-Nitro1423r for *Nitrospira*, and NTSPAf-NTSPAr for *Nitrobacter*, for heterotrophic denitrifiers as nirS (cytochrome cd1 type nitrite reductase) gene (nirScd3af-nirSR3cd).

Particularly for AnAOB, the extent of differences in primer pairs was extreme, with 24 different primer pairs in 70 studies. Further sequence alignment verified that all reported AnAOB 16S rRNA gene primers were not suitable for qPCR analysis of a biomass sample, where the AnAOB community composition is unknown, because some primers are genus specific^34^. Therefore, the usage of such primers requires previous knowledge about the AnAOB population. Undertaking a ‘rule-out’ analysis using multiple AnAOB primers is another way to avoid under-representation or false negative qPCR results. For example, one study used primer pair Amx368f-Amx820r (specific for *Ca*. Brocadia anammoxidans and *Ca*. Kuenenia stuttgartiensis) for qPCR based quantification of their AnAOB population^35^. However, the same study reported the presence of *Ca*. Brocadia anammoxidans, *Ca*. Kuenenia stuttgartiensis and *Ca*. Jettenia. This difference indicates an under-representation of the AnAOB population based on qPCR results.

### Influence of primer selection on next-generation 16S amplicon sequencing

**Figure 3:**
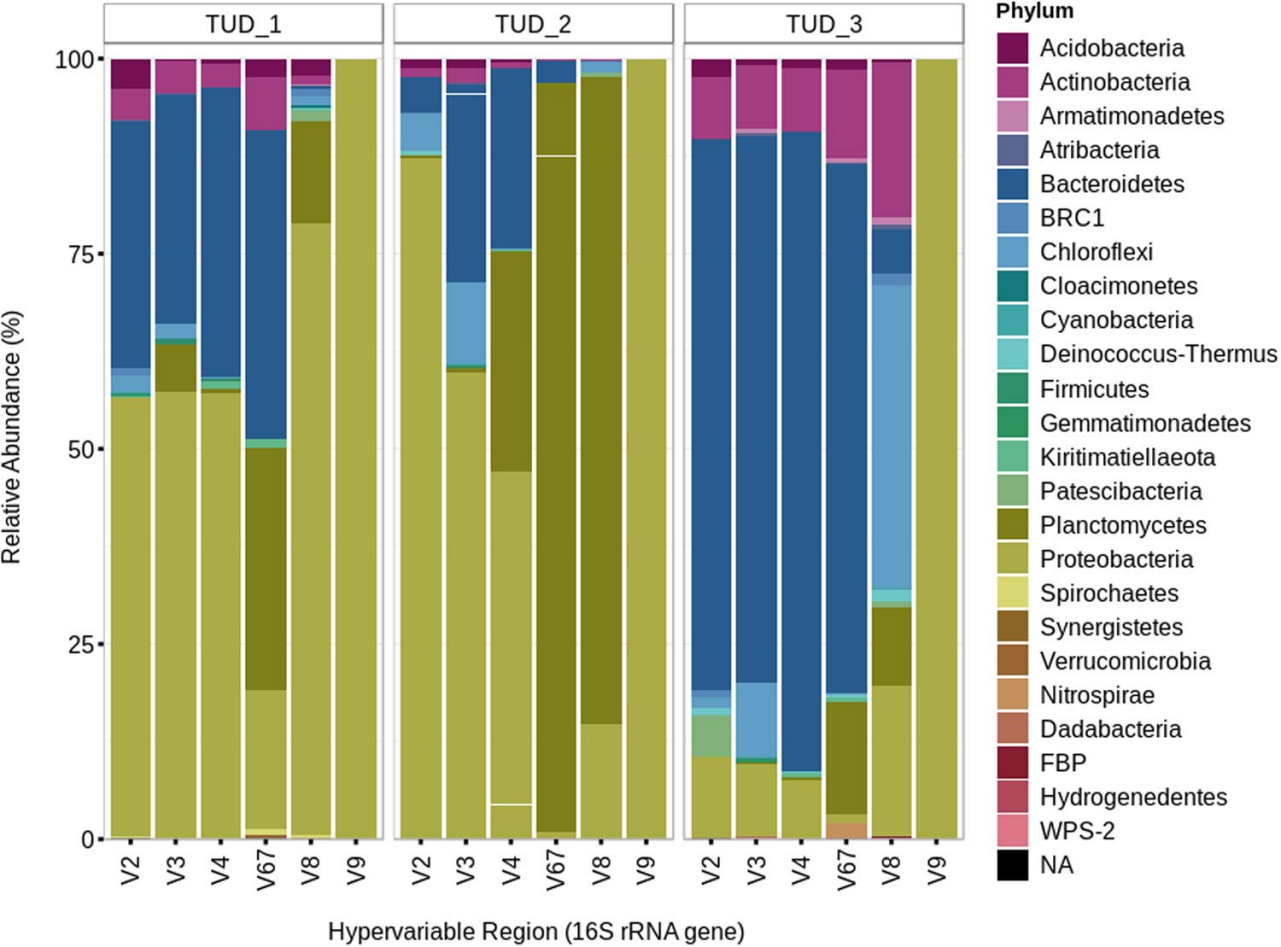
Relative abundance profiling of three samples: (1) TUD_1, (2) TUD_2, and (3) TUD_3, based on the 16S rRNA amplicon sequencing, targeting multiple hypervariable regions of 16S rRNA gene.

Although a number of reports had revealed that primer choice introduces biases in 16S amplicon sequencing^19,36–38^, no study is available yet that specifically looked at the extent of primer selection and its influence on determining the microbial communities in PNA systems. Overall, very few studies have investigated the influence of primer choice in WWTP microbial ecological studies^22,33^. Therefore, this study used 16S amplicon sequencing of multiple hypervariable regions to determine the influence of primer selection on the sequencing results in different PNA biomasses. Three different samples were selected to determine if a similar variation was observable in various samples due to primer selection. Each primer pair associated with a respective hypervariable region presented significantly different (p < 0.001; Supplementary Table 1) comprehensive information of the microbial community composition (Fig. 3). Primers for the V9 regions amplified mainly Proteobacteria; Acidobacteria were more represented by the V2 region; Firmicutes by the V3 region; Chlorflexi by the V3 and V8 regions; Bacteriodetes by the V2, V3, V4 and V6–7 regions; Nitrospira by the V6–7 region; and Planctomycetes by the V4, V6–7 and V8 regions. This implies that primers significantly influence the profiling of the total community composition. The experimental results were in consensus with the *in-silico* analysis conducted on known primers.

Some studies even made suggestions about which hypervariable region primers to use to capture certain microbial groups. Guo *et al*.^33^ suggested using the V1 and V2 region primer pairs, whereas, Albertsen *et al*.^22^ recommended V1-V3 region primers for activated sludge. However, our results show that, at least in case of PNA biomasses, there is no general “best” primer because the influence of the primers varied between the samples (Supplementary Figure 3). For example, 16S rRNA gene primers for the V6-7 and V8 hypervariable regions over-represented the AnAOB in TUD2, only. This influence of primer also affects validation of 16S amplicon sequencing data using qPCR in PNA research (Supplementary Figure 4), which is in consensus with previous study^13^. Therefore, it is important to test different primers for the respective samples and select primer pairs from multiple hypervariable regions to attain maximum coverage of the microbial community composition.

### Influence of primer selection on quantification: relative or absolute

In wastewater engineering, molecular tools are primarily used to monitor the growth of microorganisms for better process understanding and optimization^35,39–43^. Moreover, a recent study^41^ has recommended using qPCR as a validation method for other simple quantification methods used in anammox based systems. Therefore, it is essential to understand that the primers influence the quantitative nature of the PCR based methods for relative and absolute quantification.

**Figure 4:**
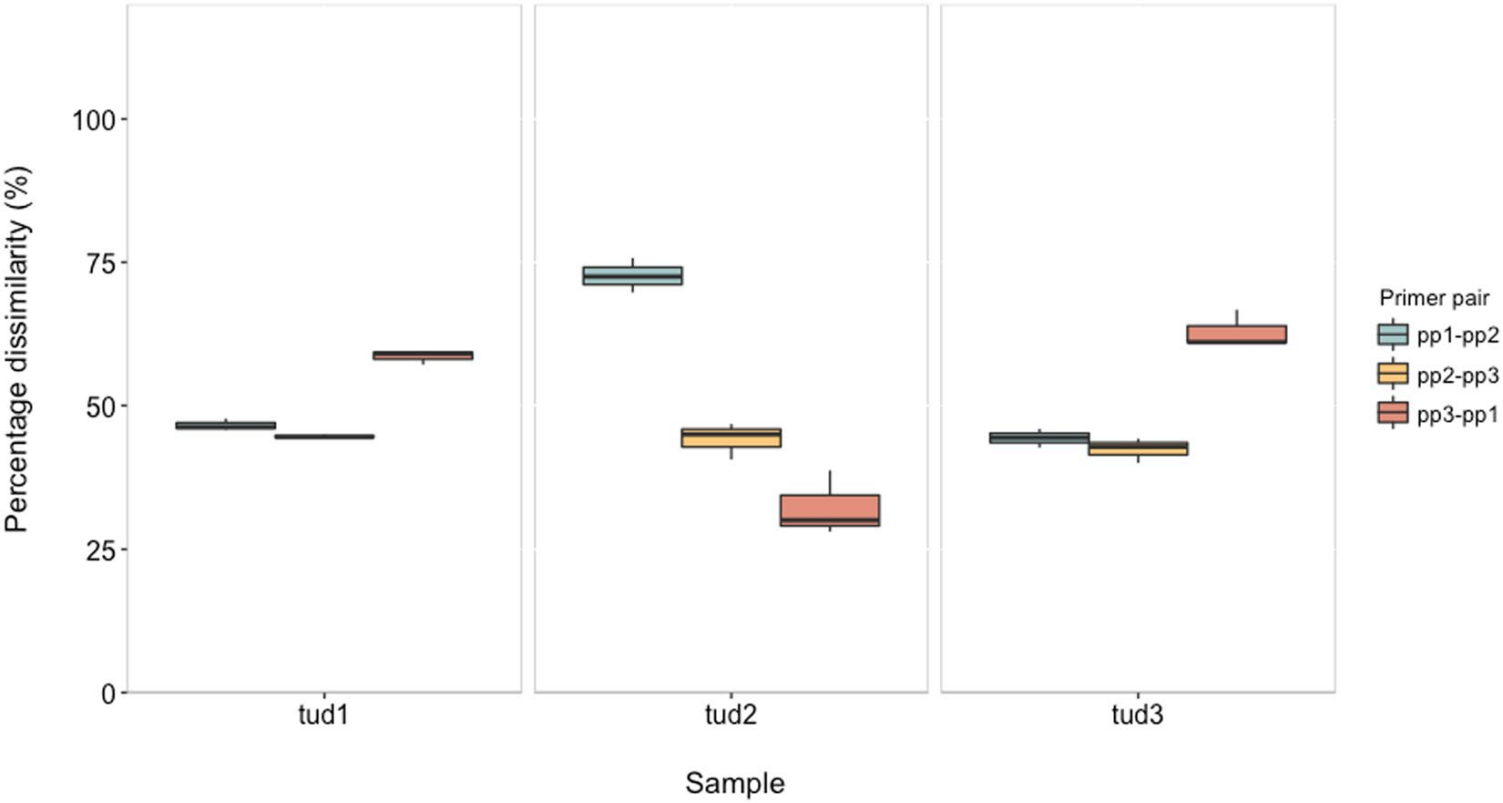
Percentage dissimilarity based on qPCR between absolute abundance of eubacterial population measured using three different primer pairs: (1) pp1 (1055f-1392r), (2) pp2 (338f-518r), and (3) pp3 (341f-543r). In legend pp1-pp2: percentage dissimilarity between pp1 and pp2, pp2-pp3: percentage dissimilarity between pp2 and pp3, pp3-pp1: percentage dissimilarity between pp3-pp1 (percentage dissimilarity was measured between a range of 0–100%, the higher the percentage greater the difference between measured absolute abundance).

Absolute quantification, based on qPCR, was performed using the three most frequently used EUB primer pairs found by the literature survey. We conducted one-way ANOVA to assess the impact of the respective primer pair on the sample. The one-way ANOVA revealed high significance of primer pair on the measured EUB microbial groups (Supplementary Table 2). However, the effect of the choice of a particular primer pair varied with the sample. Figure 4 shows the percentage dissimilarity between the absolute copy numbers of 16S rRNA genes. Dissimilarity was measured at a scale of 0–100%, the higher the percentage, the greater the difference between the measured absolute abundance. We calculated the dissimilarity based on the abundance difference between the respective eubacterial primer pairs for every sample. The percentage dissimilarity for sample TUD1 was in a range of 45 to 60%, whereas, it was between 25 and 75% for TUD2. In case of TUD3 dissimilarities were in a range of 45 to 65%. These results underline how dramatically absolute quantification data varies depending on the primers and due to variations in 16S rRNA gene copy numbers. Therefore, it is advisable that the abundance data for certain microbial groups should not be directly compared between different studies, unless the same set of primers was used. Also, the dissimilarity is greater between primers from different hypervariable regions of the 16S rRNA gene compared to primers belonging to the same region (Fig. 4). These findings are in consensus with another study^38^, which also reports that different primer pairs targeting the same region provide more comparable quantitative data.

**Figure 5:**
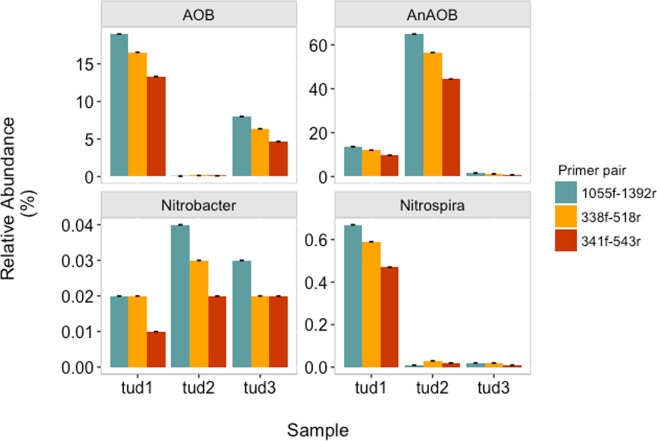
Relative abundance based on qPCR of anaerobic ammonium oxidizing bacteria (AnAOB), ammonium oxidizing bacteria (AOB), Nitrobacter (NOB) and Nitrospira (NOB) which is normalized to the abundance of total eubacteria (EUB), measured using three different primer pairs targeting two different hypervariable regions; error bar represents the standard deviation between qPCR technical triplicate runs.

In the above section, we already showed, how the relative abundance will vary in 16S rRNA amplicon sequencing data depending on primer selection. We also investigated, if similar inconsistencies occur in the relative abundances calculated from qPCR data. Depending on the 16S rRNA EUB primer pairs used for targeting the total eubacterial abundance (which also differed for different primer pairs, Supplementary Figure 5), the calculated relative abundance of AnAOB, AOB and NOB (Nitrobacter and Nitrospira) varied for all the samples (Fig. 5). After normalization of the absolute abundance of AnAOB with the absolute abundance of total eubacteria, the relative abundance varied between 10 and 15% between the three primer pairs for sample TUD1; 20–60% for sample TUD2; and 1–5% for TUD3. Based on *in-silico* analysis, the coverage of both primer pairs of region V3 – V4 for AnAOB is low, which explains the observed low relative abundance of AnAOB for primer pairs pp2 and pp3. Similar variations arose from the calculated relative abundances of AOB and NOB (Fig. 5).

PNA research, employing qPCR methods, emphasizes the quantification of the key microorganisms, based on either specific primers targeting 16S rRNA genes or functional genes, rather than the total bacterial population. However, it is a general practice to report results as relative abundance (i.e., the fraction of the total eubacterial population) in qPCR based studies^12,13,39^. Based on our results, the relative abundance approach is not advisable for PNA systems, irrespective of sample type and target microorganisms.

## Discussion

Designing a good pair of primers for qPCR is a critical factor – often highlighted in previous studies^44,45^. Therefore, primer designing has drawn much attention but mainly focused on the re-evaluation and design of new primers for specific microbial groups^21,43,45–47^ present in the PNA biomass. The rapid integration of the eco-physiological approach to study PNA systems has caused a backlog in mechanistically understanding the influence of primers on microbial ecology data. Additionally, there is a lack of guidance for the correct interpretation of such data.

In comparison to ecological diversity surveys, the objective of qPCR and/or 16S rRNA amplicon sequencing in wastewater engineering is different. The information generated serves as the basis for reactor operation and optimization, which demands comparability of quantitative data between different PNA studies. However, this is not possible unless analytical methods share common protocols^45^. Based on our results and previous literature, there is no single best primer pair, which can be recommended for PNA systems. Therefore, we recommend using a combination of multiple primer pairs. In addition, we need a best practice approach that can improve data interpretation and further simplifies the comparison of the results of different studies, in addition to following the MIQE guideline^24^.

**Figure 6:**
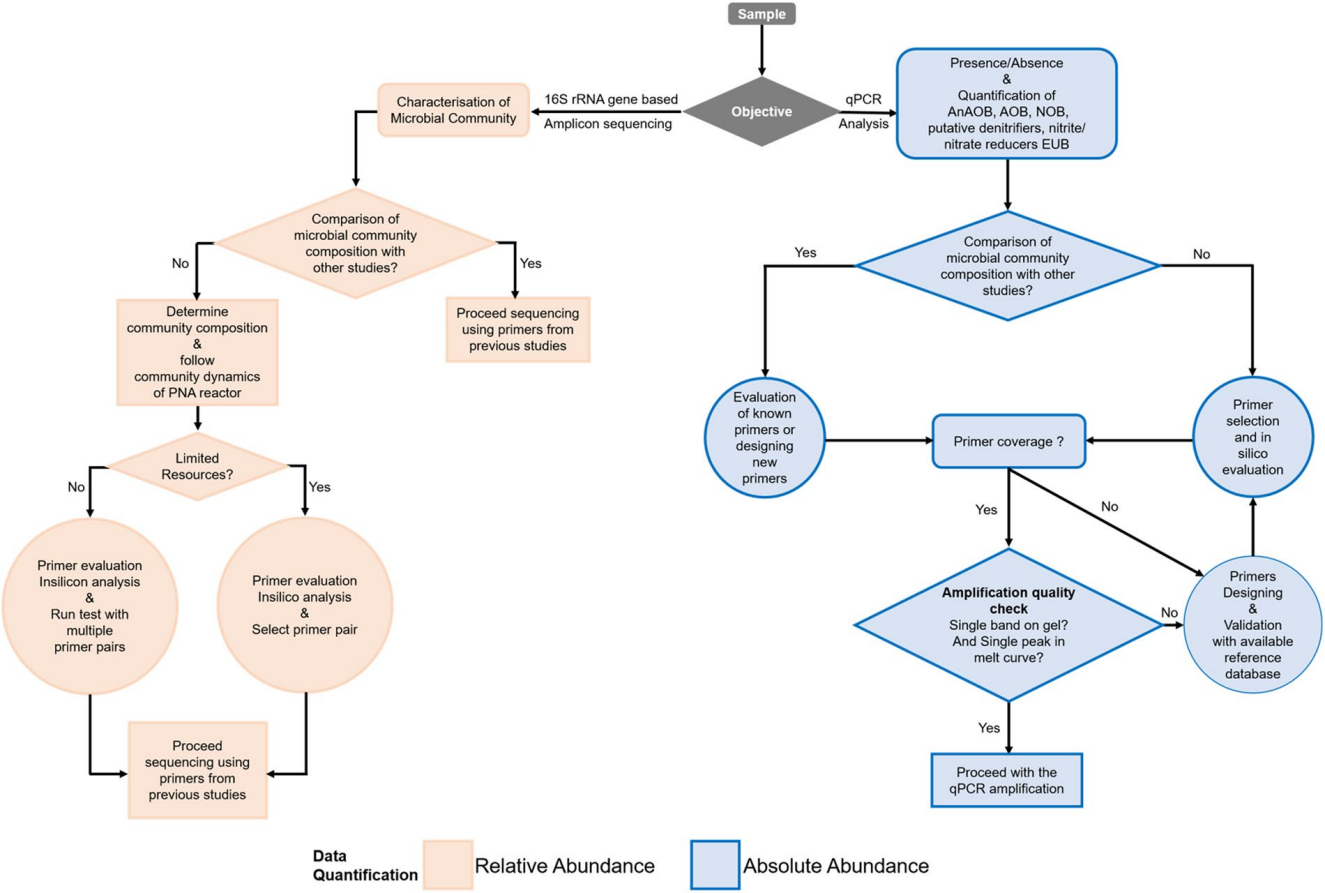
Decision tree framework for qPCR analysis and 16S amplicon sequencing for PNA systems.

Figure 6 presents a decision tree framework based on our literature assessment and experimental results. Before setting up a PCR-based approach, it is important to clarify which question needs to be answered: (1) Is the target microorganism present in the reactor system? (2) How many different microorganisms (species richness) are present in the reactor system? or (3) How many of each group of microorganisms (species evenness) are present? If the research objective is established, we suggest deciding whether the results should be provided as relative abundance using 16S rRNA based amplicon sequencing or absolute abundance using qPCR (as represented in Fig. 6 with different colors).

Apart from defining the ecological question, it is also important to define whether the focus of the study is (1) to compare the results with other PNA studies, and/or (2) to study the community composition and dynamics of the PNA system. If the objective is to compare results with other PNA studies, based on growth rates and turnover, we recommend choosing the same primer pairs as used in these other studies. The use of different primers may introduce biases in community profiling (as shown in Fig. 3) and thus reduces confidence in the comparison of studies. Nevertheless, before using the reported primer pairs, always verify the quality of the PCR product (whether it is a single band or multiple bands) with gel electrophoresis, except for degenerated primers. Although gel electrophoresis might seem an old-school method, it is still the only method to verify the quality of a PCR product visually. If the focus is to study the community composition and dynamics of a PNA reactor, selected primers should be evaluated with *in silico* analysis to determine the coverage of the primer pair. It is also important to remember that the obtained results might not provide information about the whole microbial community composition. Therefore, studies focusing on temporal dynamics of the microbial community should interpret results relative to a reference sample belonging to the same PNA reactor. The mentioned set of questions will help to decide which primer pairs can be used for the study. We strongly advise against the normalization of the measured abundances for AnAOB, AOB, NOB and putative heterotrophs to the total eubacterial abundance. This interpretation of results might lead to false positive or false negative results. For instance, we observed different abundances of AnAOB based on normalized data (Fig. 5).

Regardless of the objective, an *in silico* analysis for choosing the appropriate primer pair is an essential step due to the range of primer sets of the respective target group. There are also non PCR-based methods like FISH offering complementary information, which can also be useful to design new primers and probes. Despite the hurdles being stated here, PCR based methods are positively acknowledged to determine microbial composition in PNA systems because they are sensitive and fast techniques^48^. A wise choice of primers and the mentioning of the information about the coverage of the primers will then further boost the confidence in such results.

## Materials and Methods

### Scientific literature assessment

**Figure 7:**
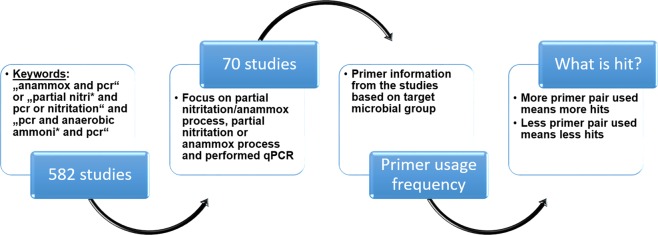
Schematic showing the approach used to extract PNA studies, which were used for the assessment.

To evaluate previous studies, we conducted an internet search using the Web of Science platform (v.5.27.2) by Thomson Reuters and collected research papers using a search query with the following keywords: “anammox and pcr” or “partial nitri* and pcr or nitritation” and “pcr or anaerobic ammoni* and pcr” (Fig. 7). The use of keywords with asterisk helped to find all the studies that shared at least the same root word with the same five or six letters in the beginning. The search considered papers between 2006 and 2016 and found the total of 582 studies based on the keywords. Out of these 582 studies, 70 studies remained focusing on partial nitritation anammox (PNA), partial nitritation (PN) and/or anammox (A) reactor systems and performed qPCR analysis (Fig. 7, supplementary Table 3). Information about the type of the reactor systems that were used in these studies is provided in the supplementary material (supplementary Figure 1).

The primer information extracted from these studies was sorted based on the target microbial group and the usage frequency (hits) (Fig. 7). For every single study no hits or more than one hit is possible for each target microbial group depending on the experimental aims of the respective study. Further, *in silico* PCR analysis was performed for the 16S rRNA gene primers from the literature, targeting the total eubacterial population. *In silico* PCR analysis was performed to determine the coverage of the primer pairs, respectively. The coverage of primers was tested using the SILVA test prime function based on the version SILVA132 (https://www.arb-silva.de/search/testprime/).

Additionally, 16S rRNA gene primers were aligned using Unipro UGENE^49^ a multiplatform, open-source application as a sequence alignment tool, with 16S rRNA gene sequences chosen of representative microbial members in PNA systems.

### 16S amplicon sequencing

Biomass samples were collected from three different PNA reactors: a full-scale single stage sidestream PNA (TUD1), a lab scale single stage PNA (TUD2) and a full-scale anammox stage sidestream PNA (TUD3). Total genomic DNA was extracted using the Fast DNA Spin kit for soil (MP Biomedicals) according to a modified manufacturer’s protocol. The quality of the DNA was checked using gel electrophoresis, and the concentration was measured using a Qubit 3.0 Fluorometer (Thermo Fisher Scientific). For each sample, multiple hypervariable regions of 16S rRNA genes were amplified with the 16S Ion Metagenomics Kit™ (Thermo Fisher Scientific) by two separate PCR reactions, amplifying the V2, V4, V8 and V3, V6–7, V9 hypervariable regions, according to the kit protocol^9^. Equal volumes of V2, V4, V8 and V3, V6–7, V9 amplicons were combined. 100 nanograms of pooled amplicons were processed to the amplicon library using the Ion Xpress Plus Fragment Library Kit™, and each sample was tagged using the Ion Xpress Barcodes Adapters™ (Thermo Fisher Scientific), according to the manufacturer’s protocol. Each sample was adjusted to a 10 picomolar concentration. All three samples were pooled, in equal volumes, and processed with One-Touch 2 and One-Touch ES systems (Thermo Fisher Scientific) according to the manufacturer’s instructions.

Sequencing was performed on the Ion Torrent (ION Torrent Ion S5) using the 400-bp kit and 530 chip. Base calling and run demultiplexing were conducted by Torrent Suite version 4.4.2 (Thermo Fisher Scientific) with default parameters. The Ion Reporter^TM^ software (Thermo Fisher Scientific) is a bundle of bioinformatics tools, which uses QIIME ver. 1.9.1 to process 16S metagenomic data^50^. QIIME was implemented for separating sequences based on their respective targeted regions and OTU (operational taxonomical unit) picking with its default settings. Overall, the *de novo* clustering of OTUs was done with 97% identity, corresponding to species level. The sequences were classified based on the taxonomy in the Silva database (97% confidence threshold, version 132)^51^. The sequencing data were analyzed in R, using ggplot2 (v0.9.3.1) and two-way analysis of variance (ANOVA) to test significance of the results.

### Quantitative PCR

Total genomic DNA was extracted from biomass samples using the Fast DNA Spin kit for soil (MP Biomedicals). DNA concentration and its integrity were analyzed using Qubit 3.0 Fluorometer with Qubit dsDNA HS kit (Thermo Fisher Scientific). The abundance of total bacterial abundance (EUB) was quantified targeting the V3–4 region of the 16S rRNA gene (primer pair 338f-518r and primer pair 341f-543r) and V7–8 region (primer pair 1055f-1392r). The abundance of AOB, AnAOB, and NOB was quantified targeting the ammonia monooxygenase (amoA) gene (primer pair amoA1f/amoA2r), and the 16S rRNA genes for AnAOB (primer pair Amx809f-Amx1066r), for Nitrobacter (primer pair Nitro1198f-Nitro1423r), and for Nitrospira (primer pair NSR1113f-NSR1264r). qPCR analysis was performed for each sample and primer pair as technical triplicate runs. Each qPCR run was then performed in triplicates for a 25 µL reaction mixture containing 12, 5 µL of PerfeCTa SYBR® Green SuperMix 2X (QuantaBio), 0, 5 µL of each primer, 5 µL of DNA (5 ng/µL) and PCR grade water. Thermal profiles for each primer pair are available in the supplementary information (Supplementary Table 4). The qPCR abundance data were analyzed in R, using ggplot2 (v0.9.3.1) and one-way ANOVA.

Percentage dissimilarity was calculated to determine the impact of primer pair on the measured total eubacterial abundance. The percentage dissimilarity attributed to each primer pair, was calculated using a similarity percentage (SIMPER) analysis. The dissimilarity between the measured absolute abundance using three different primer pairs (pp.1 1055f-1392r, pp.2 338f-518r, pp.3 341f-543r) is reported as a percentage.

## Supplementary information

Supplementary Information

## Data Availability

OTU representative sequences were submitted to the GenBank under the accession numbers MH682261 - MH683001.
