# Supplementary material for: On resolving ambiguities in microbial community analysis of partial nitritation anammox reactors
Source: Sci Rep. 2019 May 6;9:6954. doi: 10.1038/s41598-019-42882-8 (PMC6502876; doi:10.1038/s41598-019-42882-8)
Supplement: Supplementary file 1 — Supplementary Information [file 41598_2019_42882_MOESM1_ESM.docx]

Supplementary Information to

On resolving ambiguities in microbial community analysis of partial nitritation anammox reactors

Laura Orschler^1^, Shelesh Agrawal^1, *^, Susanne Lackner^1^

*^1^ Technische Universität Darmstadt, Institute IWAR, Chair of Wastewater Engineering, Franziska-Braun-Straße 7,64287 Darmstadt, Germany*

*** *E-mail: s.agrawal@iwar.tu-darmstadt.de, Phone: +49 615 116 21039, Fax: +49 615 116 20305.*

1. Types of reactor systems

In the surveyed literature, reactors with suspended biomass were the most studied group, the second most group were MBBRs, followed by MABRs and granular sludge reactors (Supplementary Figure 1). More than 80% of the suspended biofilm reactors were set up for side-stream treatment, which is comparable with the proportional distribution of the granular biofilm reactors, MBBR and MABR.

PNA reactors were the most common reactors used in all studies and studied three-times more than PN reactors. More than half of the studied reactors were operated with intermittent aeration, and only one third was operated with continuous aeration.


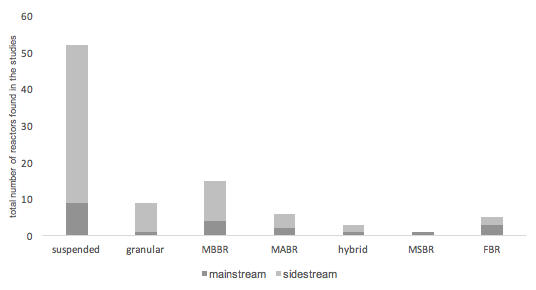

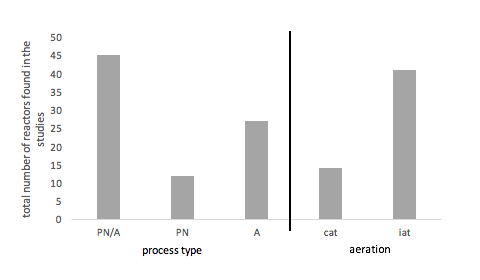


Supplementary Figure 1: Data evaluation for the reactor types divided into seven groups: suspended biomass, granular sludge, moving bed biofilm reactor (MBBR), membrane aerated biofilm reactor (MABR), hybrid, membrane sequencing batch reactor (MSBR) and fluidized bed reactor (FBR); further subcategorization into process type: partial nitritation/anammox (PNA), partial nitritation (PN) and anammox (A) and continuously aerated (cat) and intermittently aerated (iat)

1. Multiple sequence alignment

We performed alignment of the multiple 16S rRNA gene sequences associated to the known representatives of the PNA community, as reported in a previous study^1^, with the eubacterial 16S rRNA gene primer pairs found in the literature assessment (Table 1). Additionally, we aligned two Escherichia coli sequences for the verification of the primer pairs. It is important to note that the 16S rRNA gene sequences used for the alignment represented a small fraction of the total PNA community. Especially in case of heterotrophic members of the micobrial communtiy, which have wide diversity.

We evaluated eight primer pairs belonging to various hypervariable regions of the 16S rRNA gene, two for the V3/4 region, one for the V3/V5 region, two for the V4/5 region, one for the V6/7 region, one for the V7/8 region and one for the V8/9 region. Only, primer pair 1055f-1392r covered all members relevant for the PN/A process, i.e. Planctomycetes and γ-Proteobacteria (Supplementary Figure 2), however it did not cover the whole microbial community in PN/A systems e.g. Bacteriodetes. Primer pair 519f-907r do not cover any of the representative sequences. On the other hand, none of the primer pairs covered the sequence of *Cryomorpha ignava*, *Fimbriimonas ginsengisoli Gsoil 348* and Candidatus *Roizmanbacteria bacterium GW2011_GWC2_35_12 UR63_C0022*.


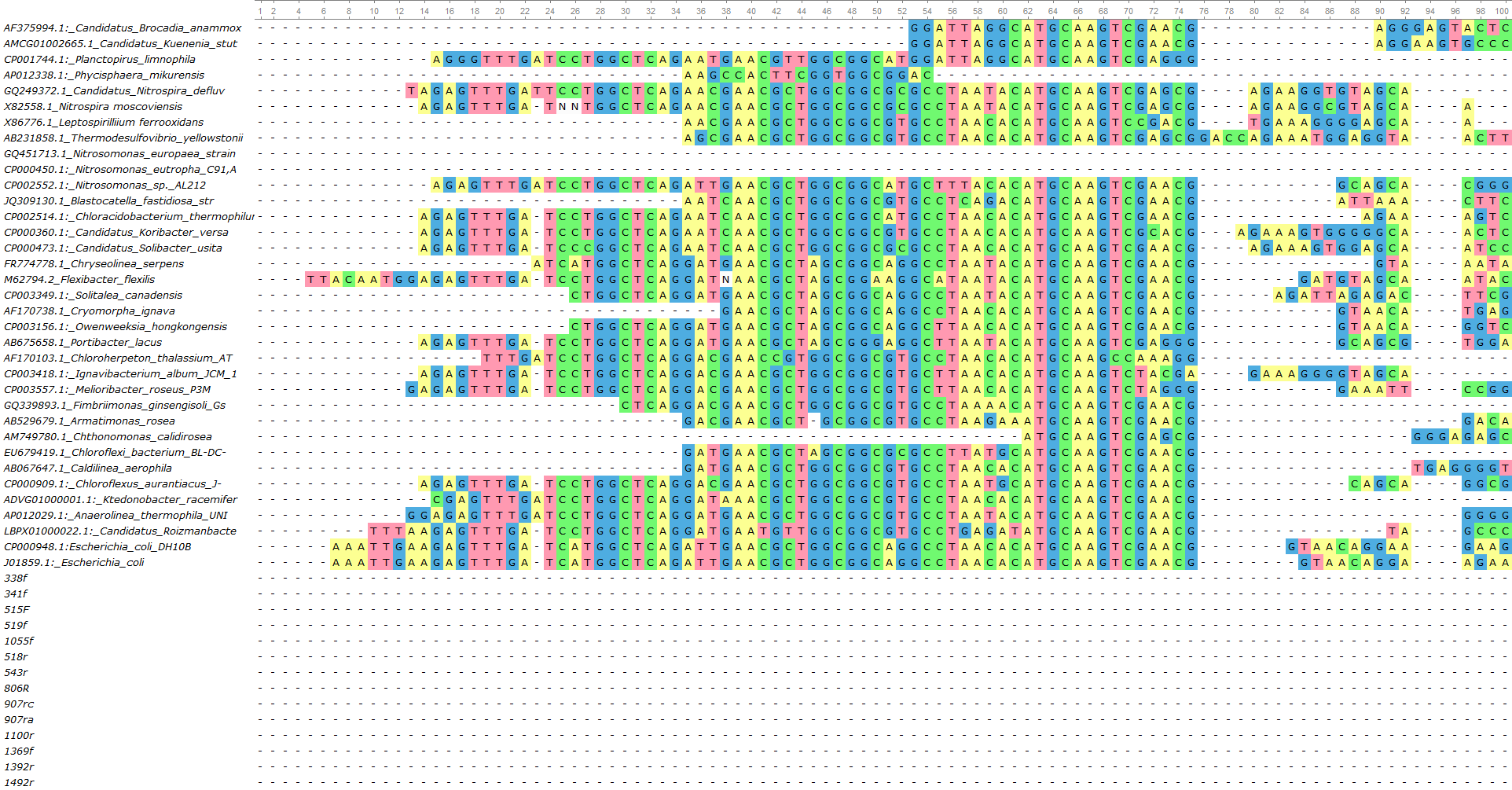

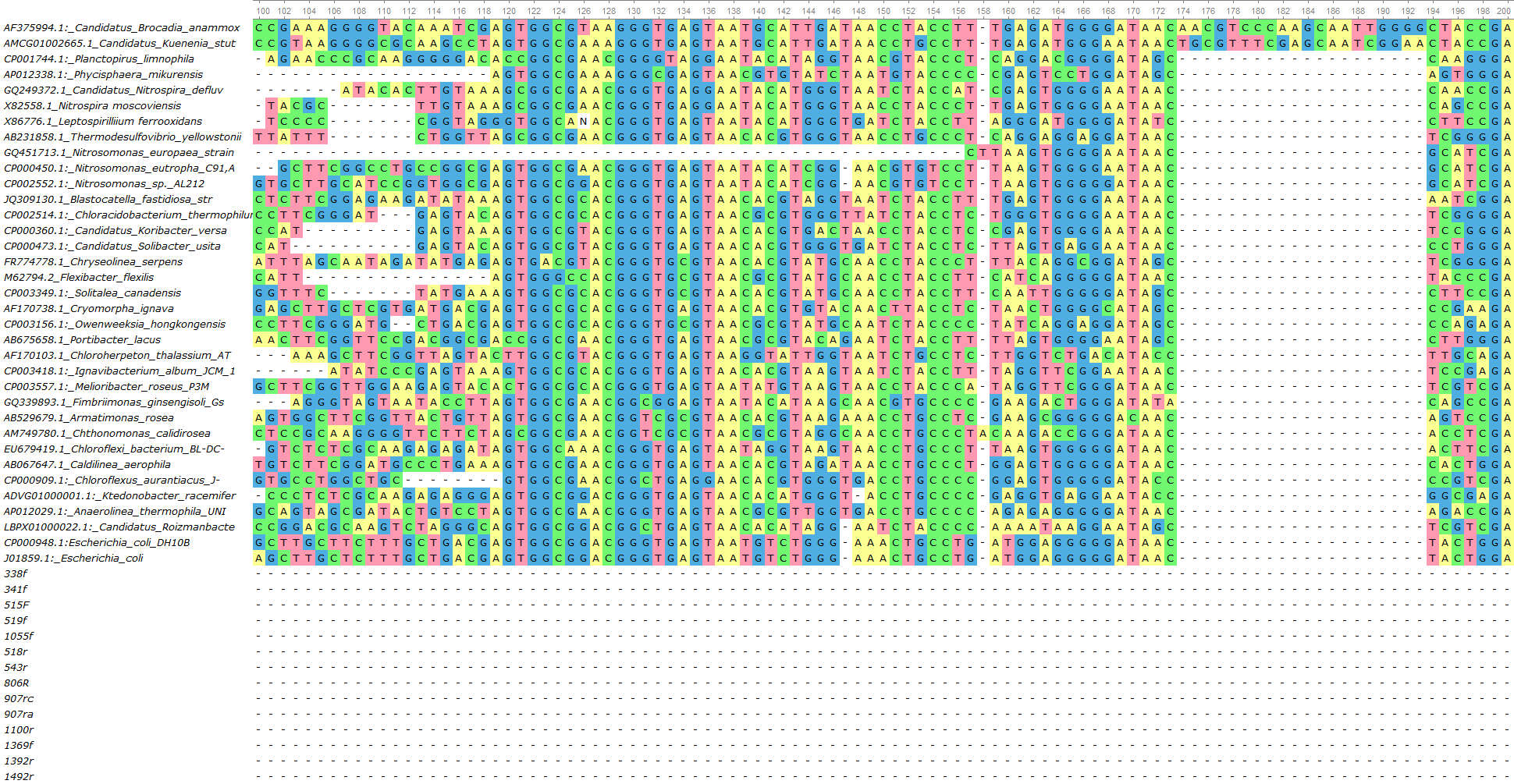

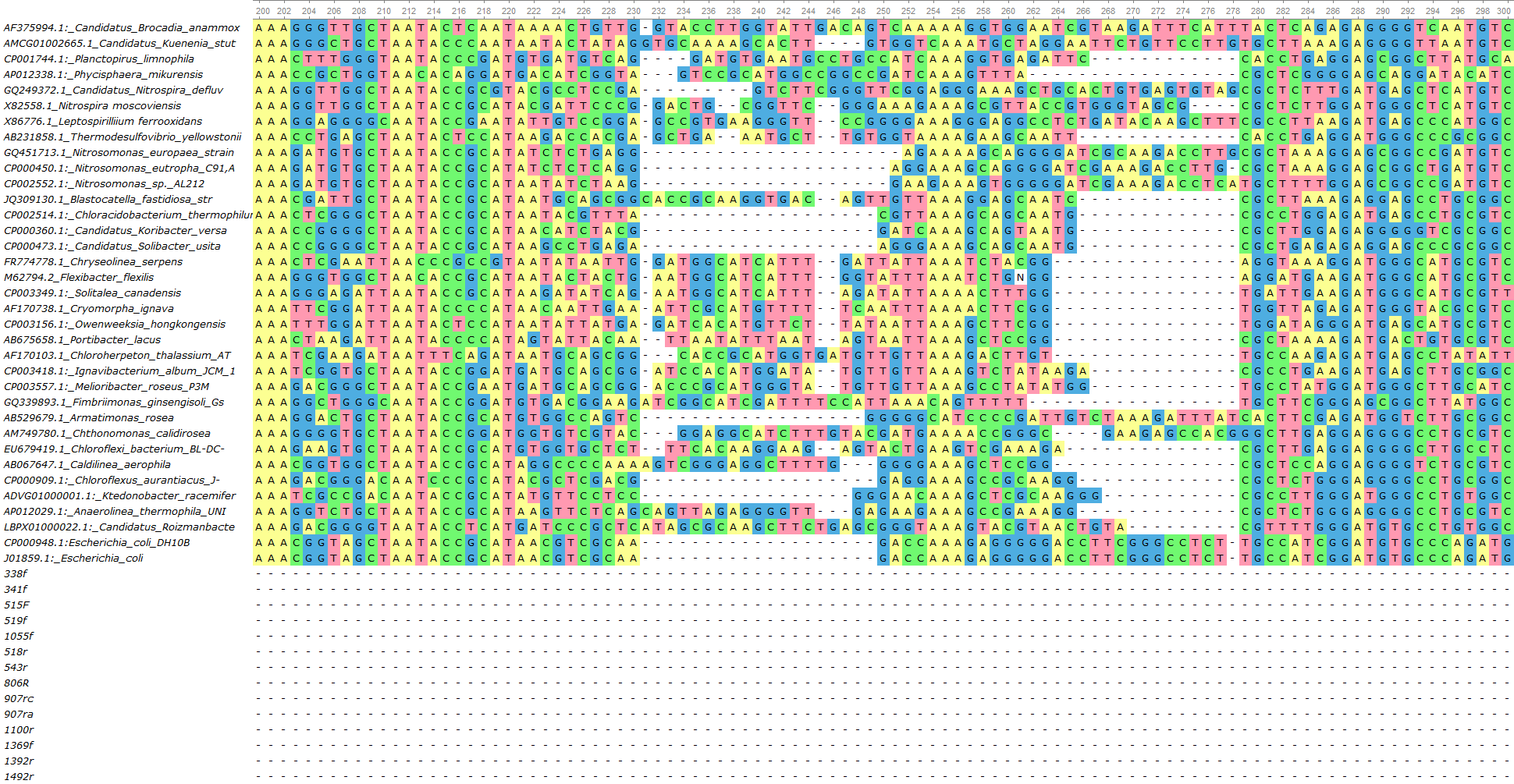

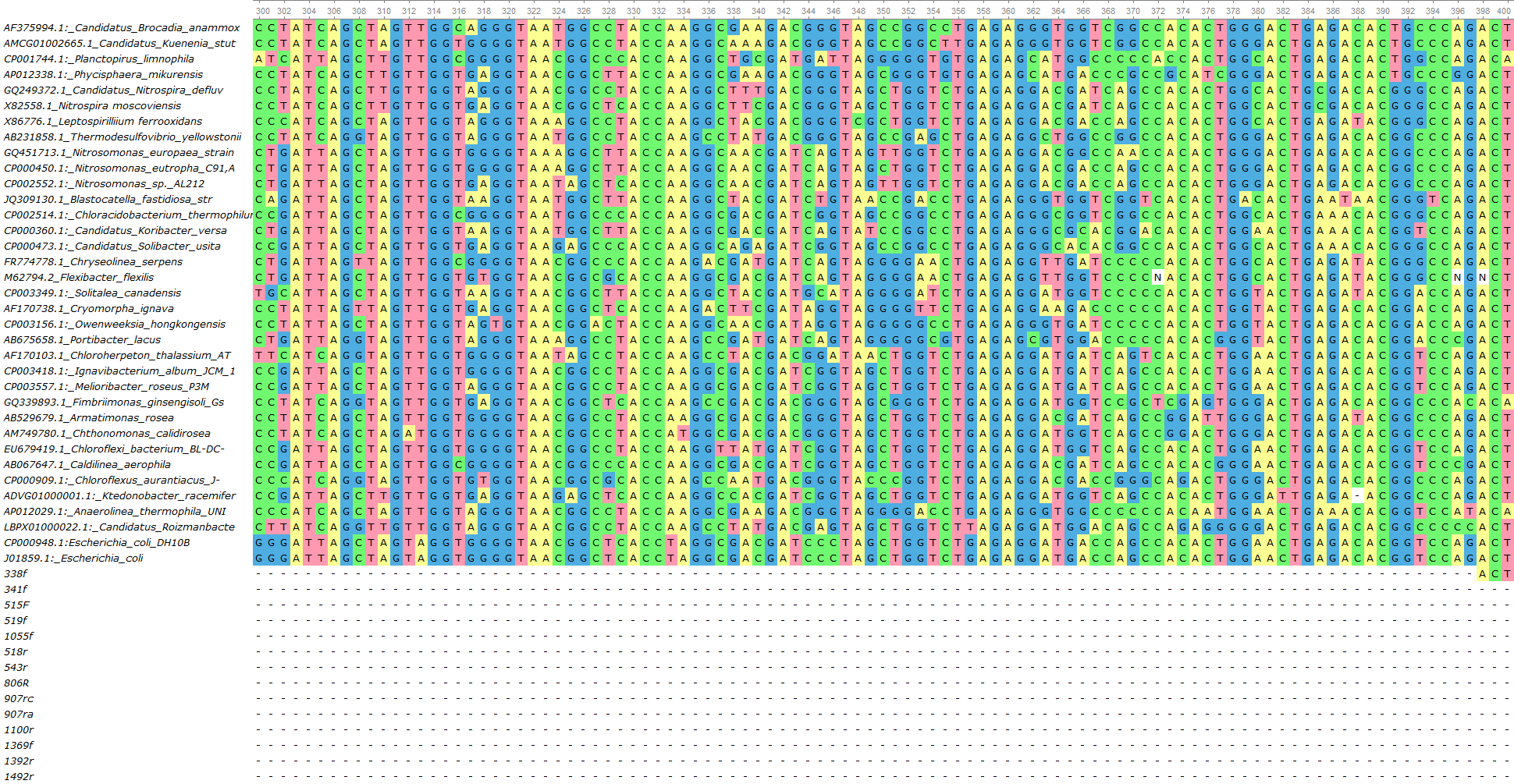

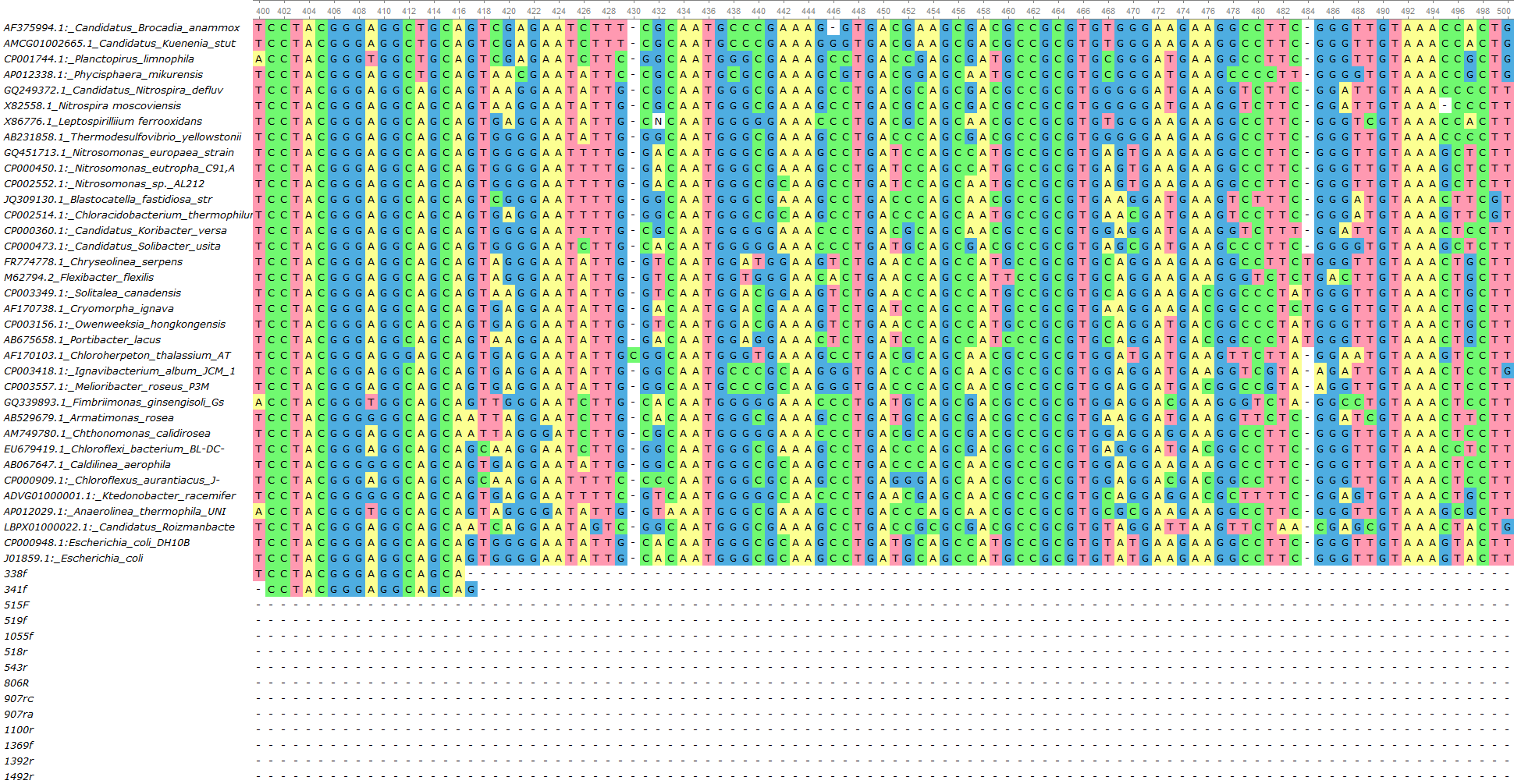

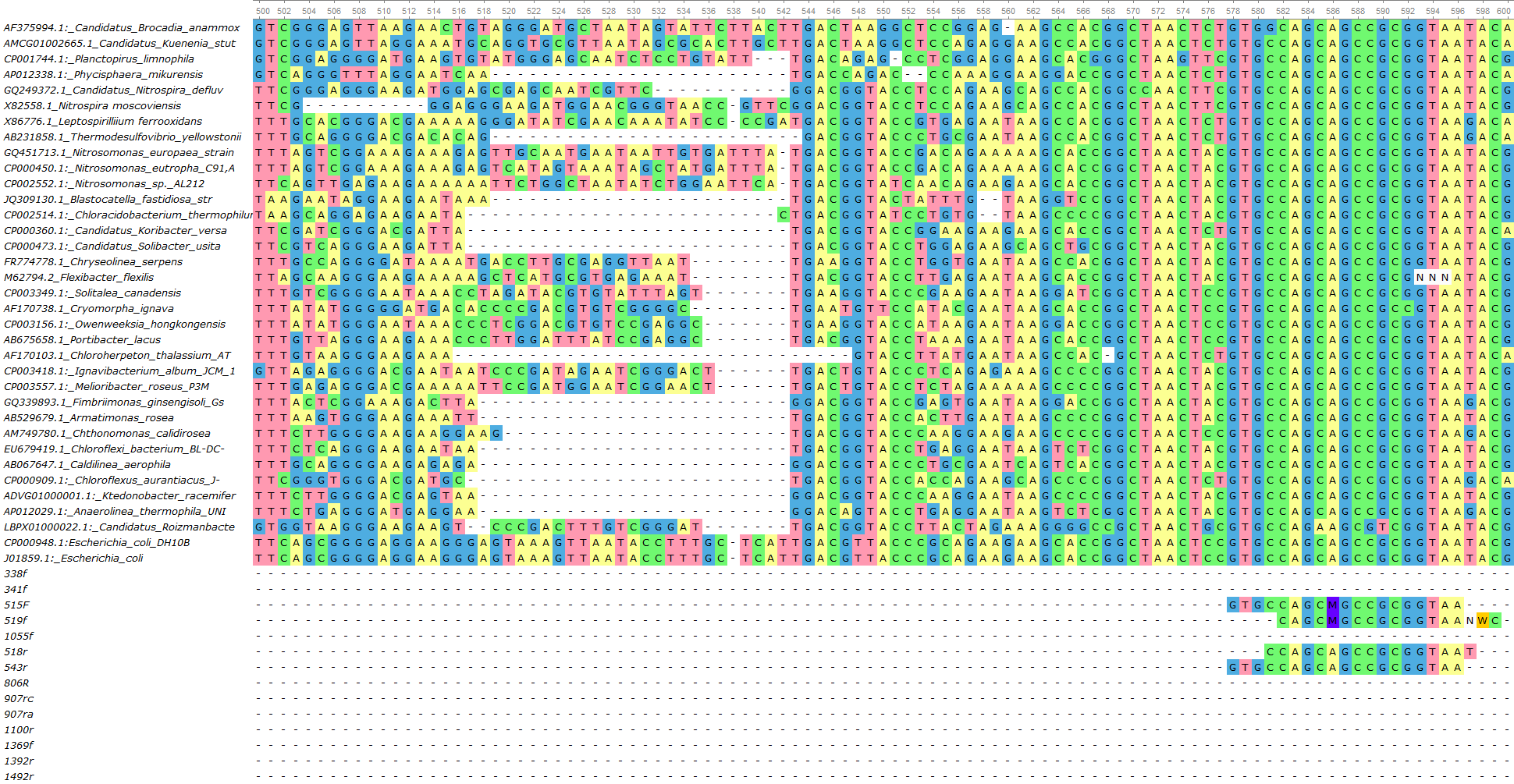

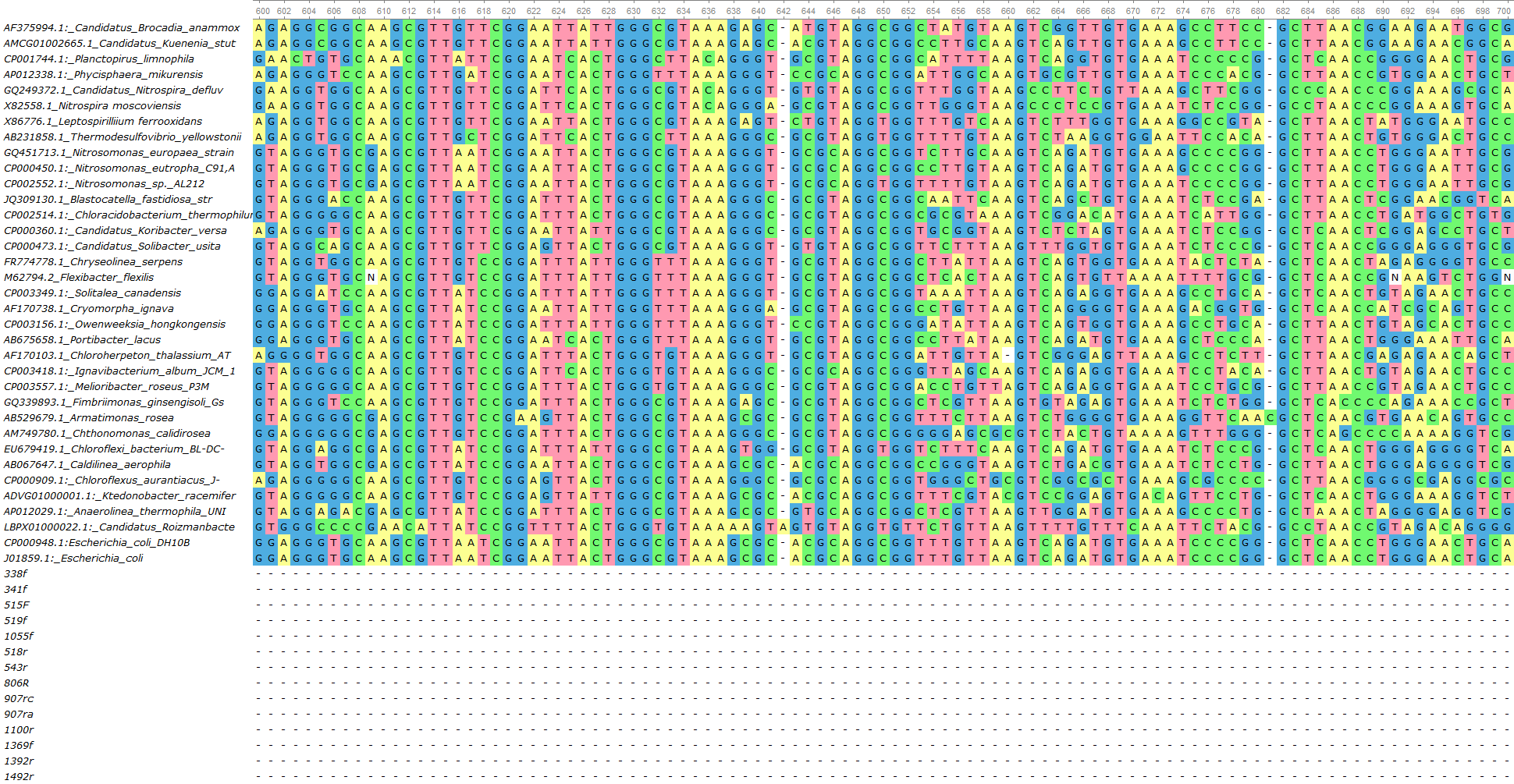

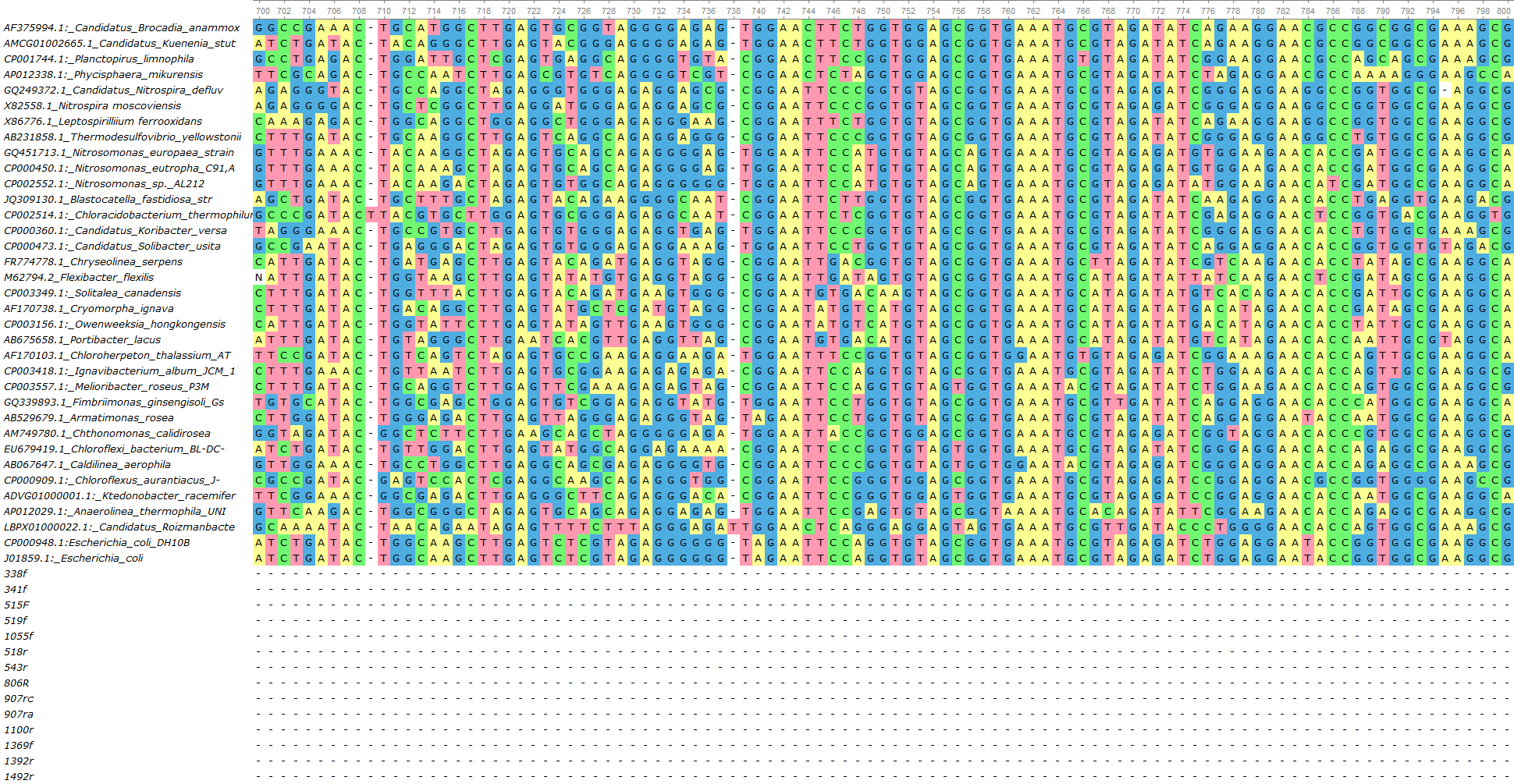


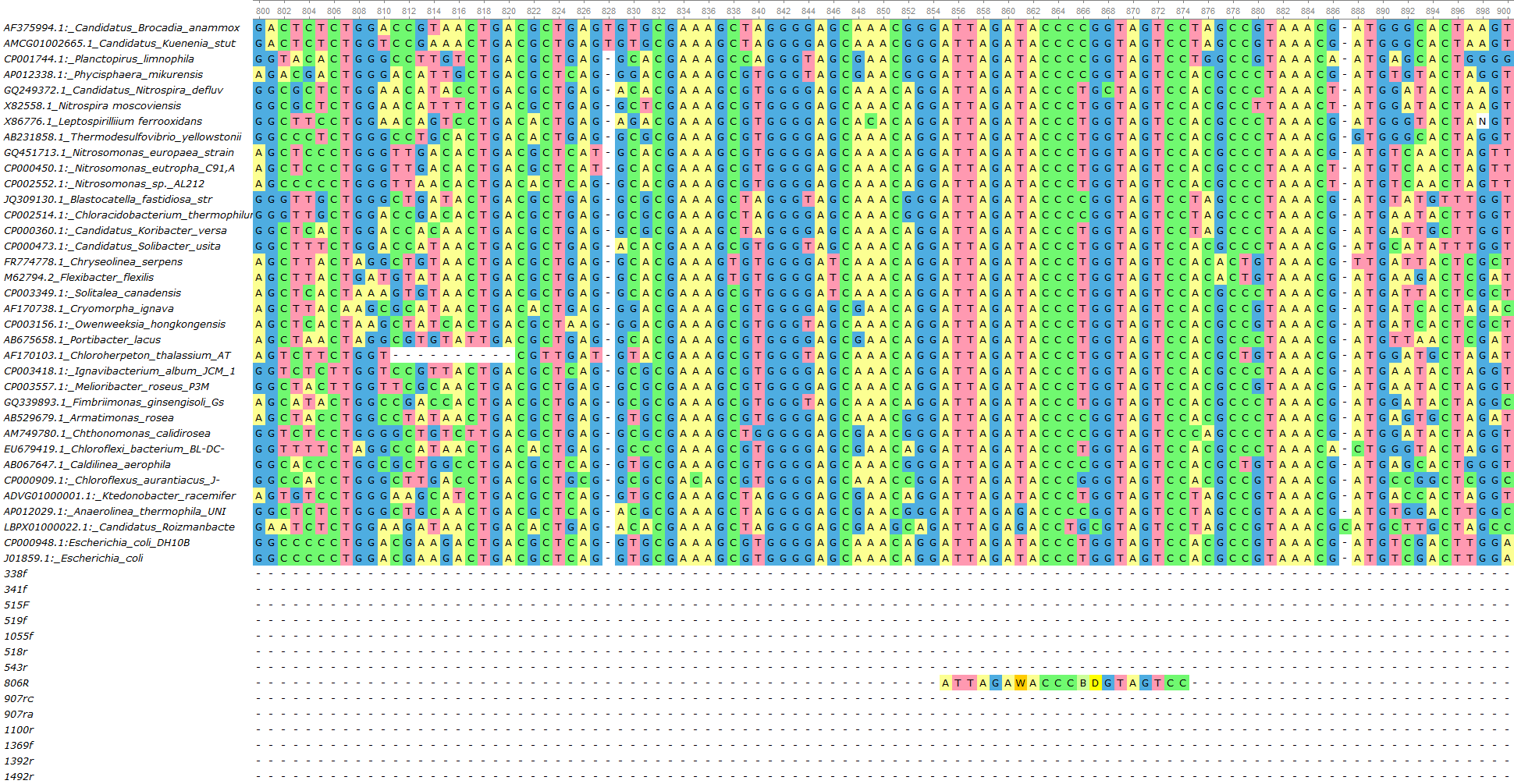


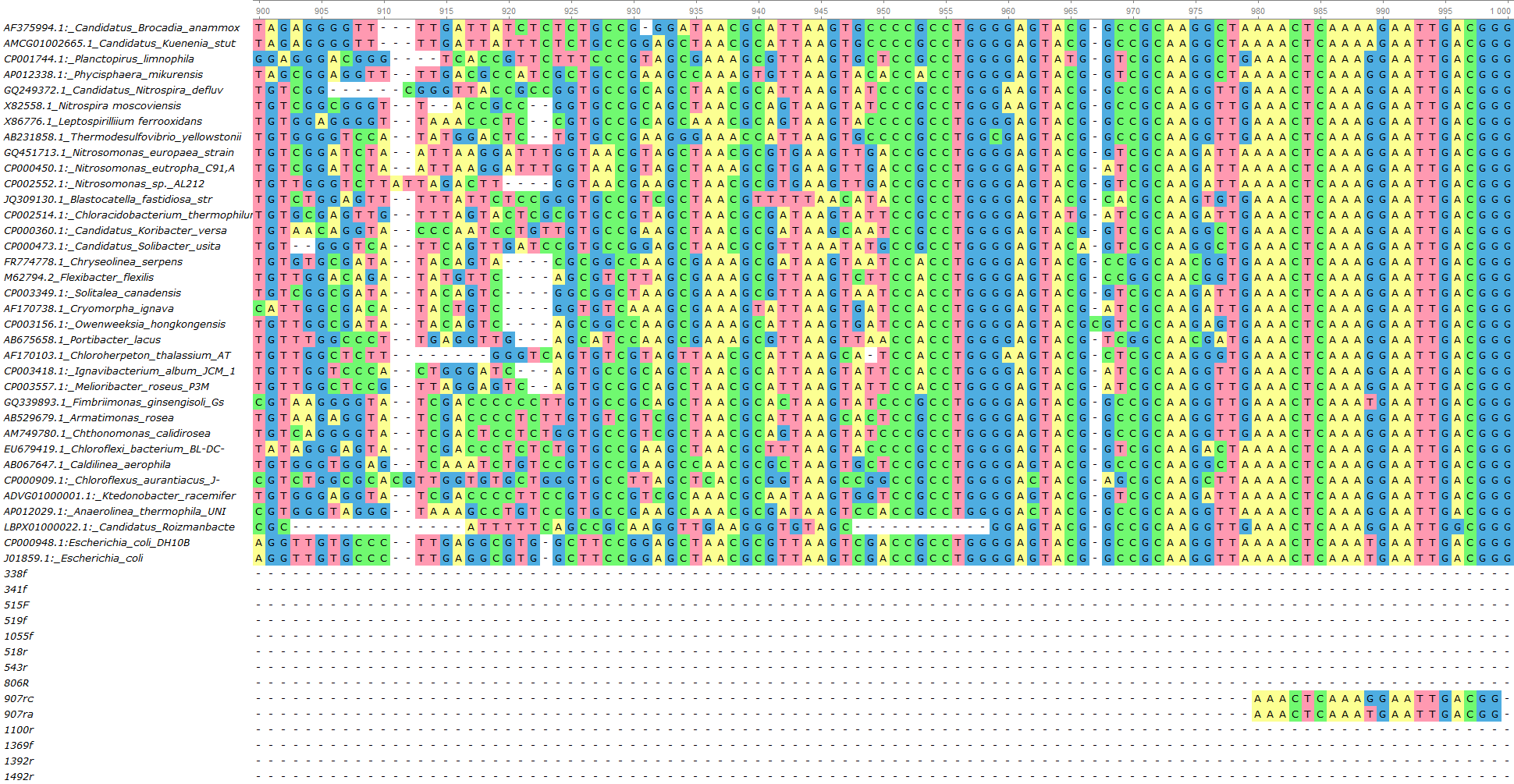


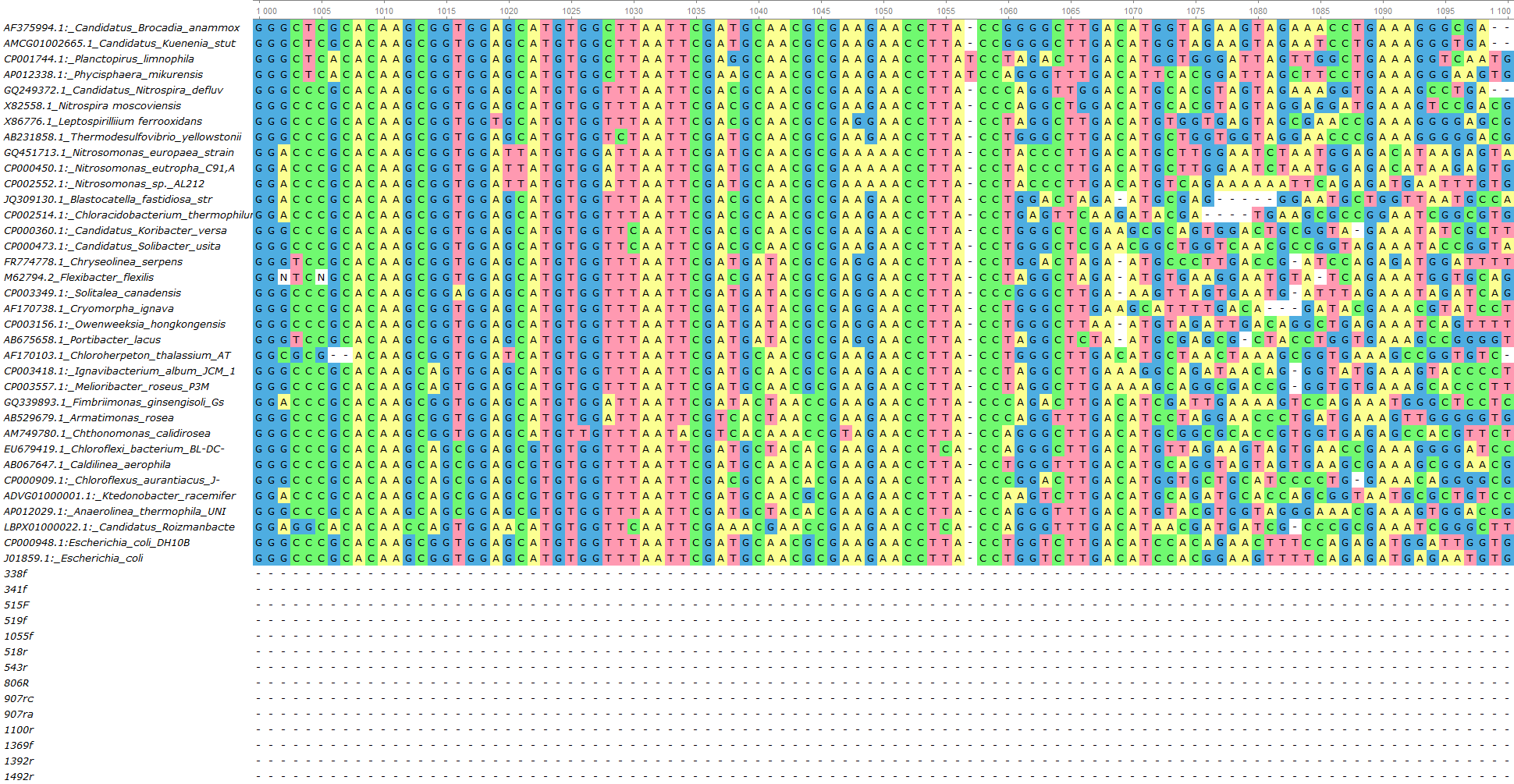


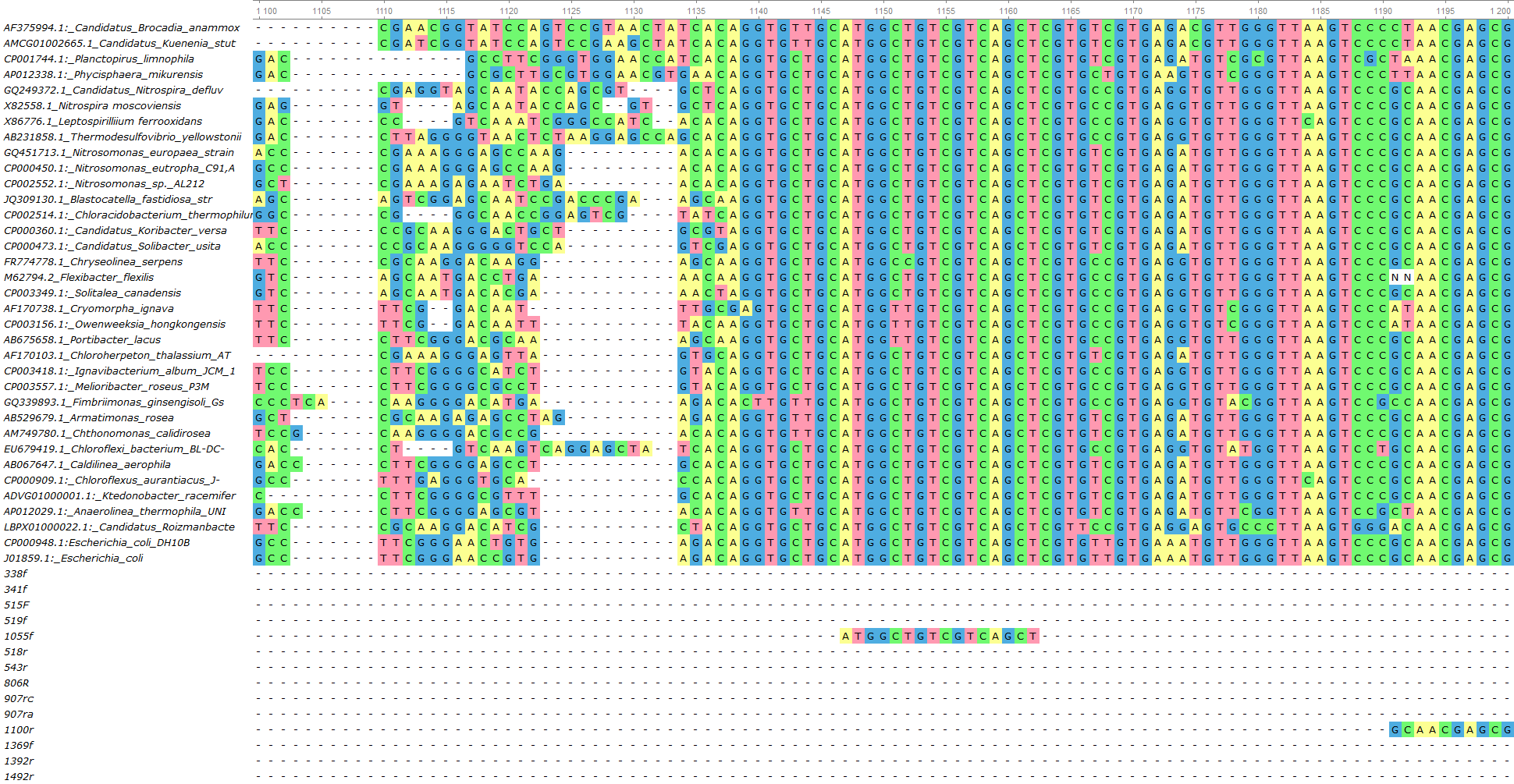


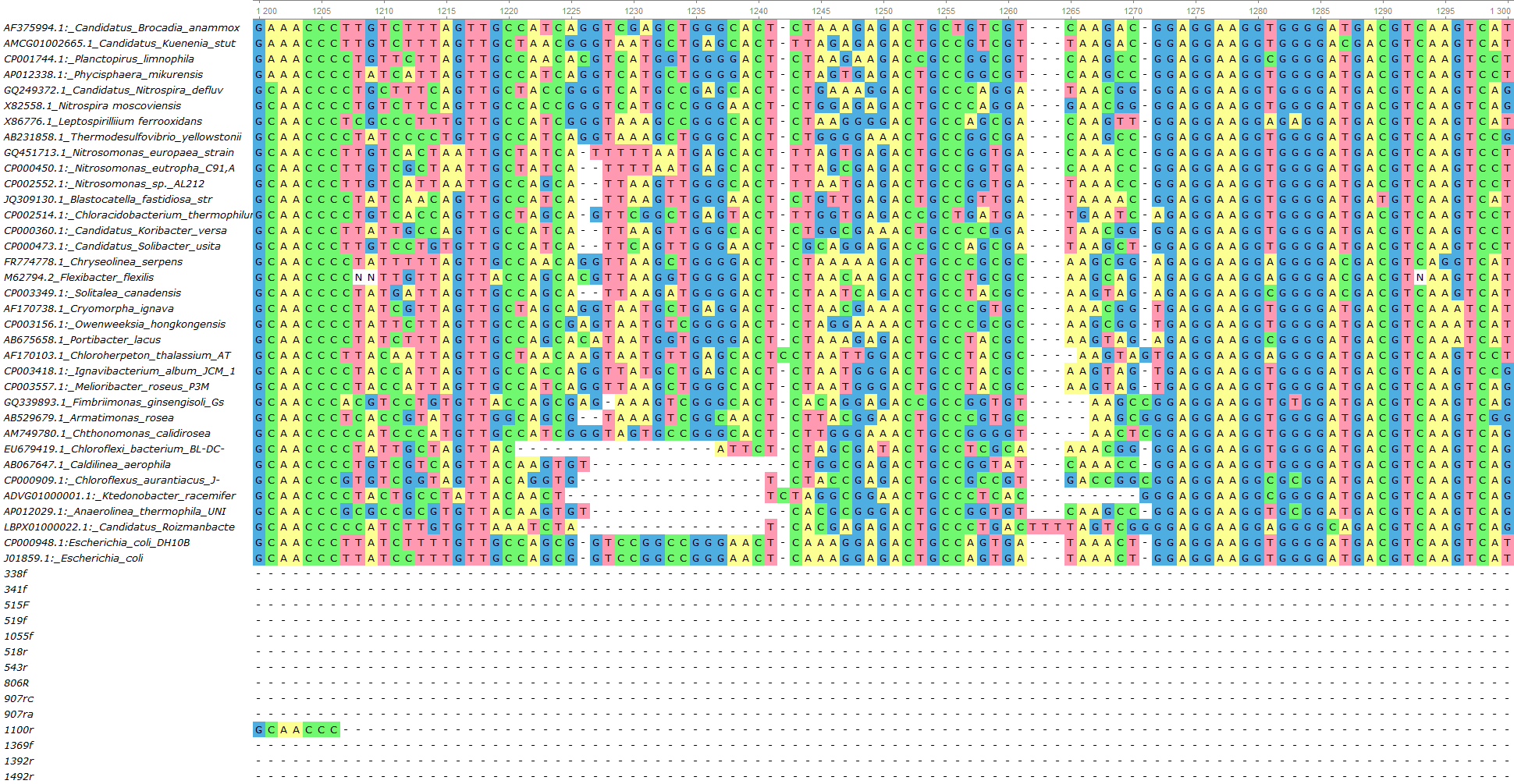

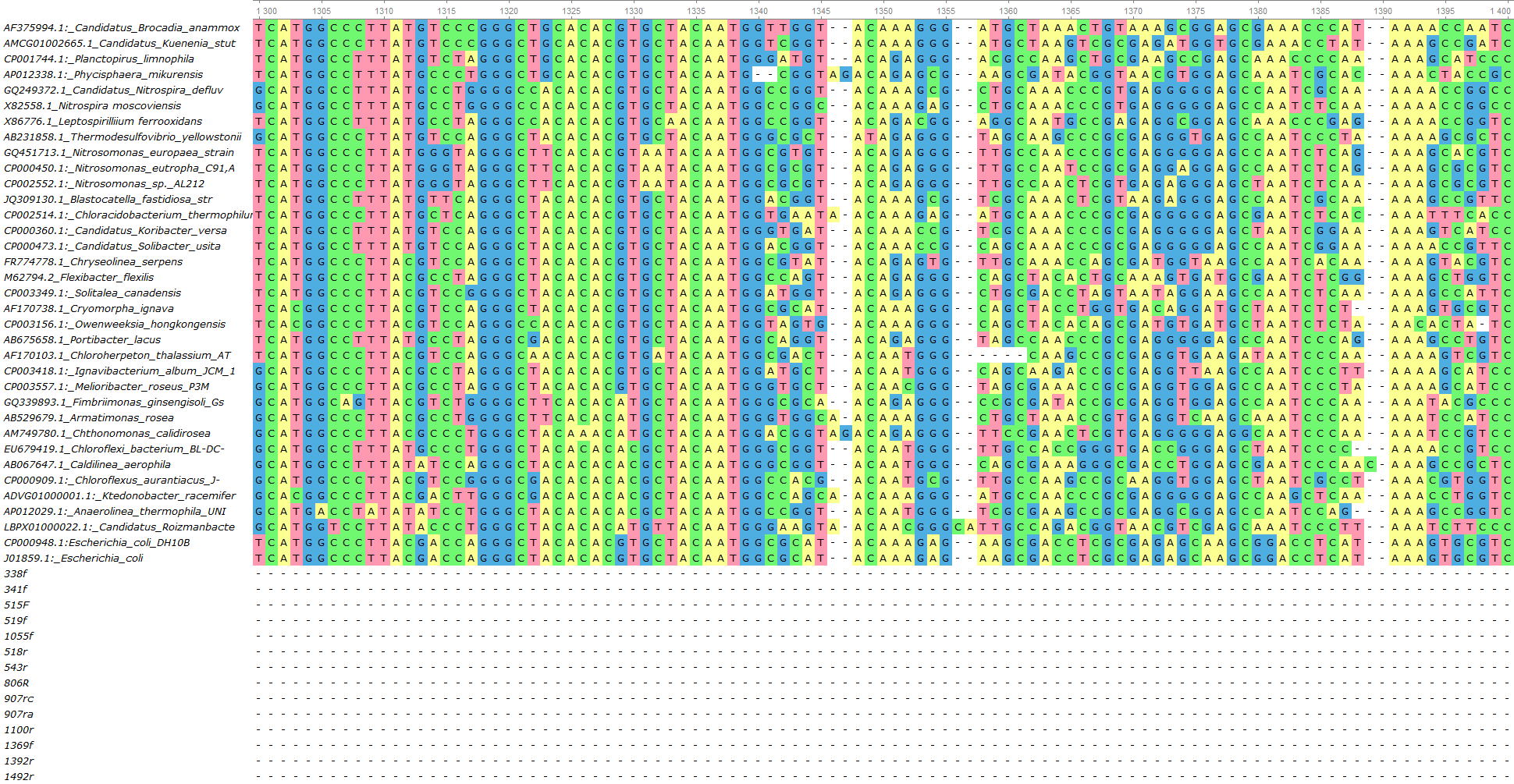

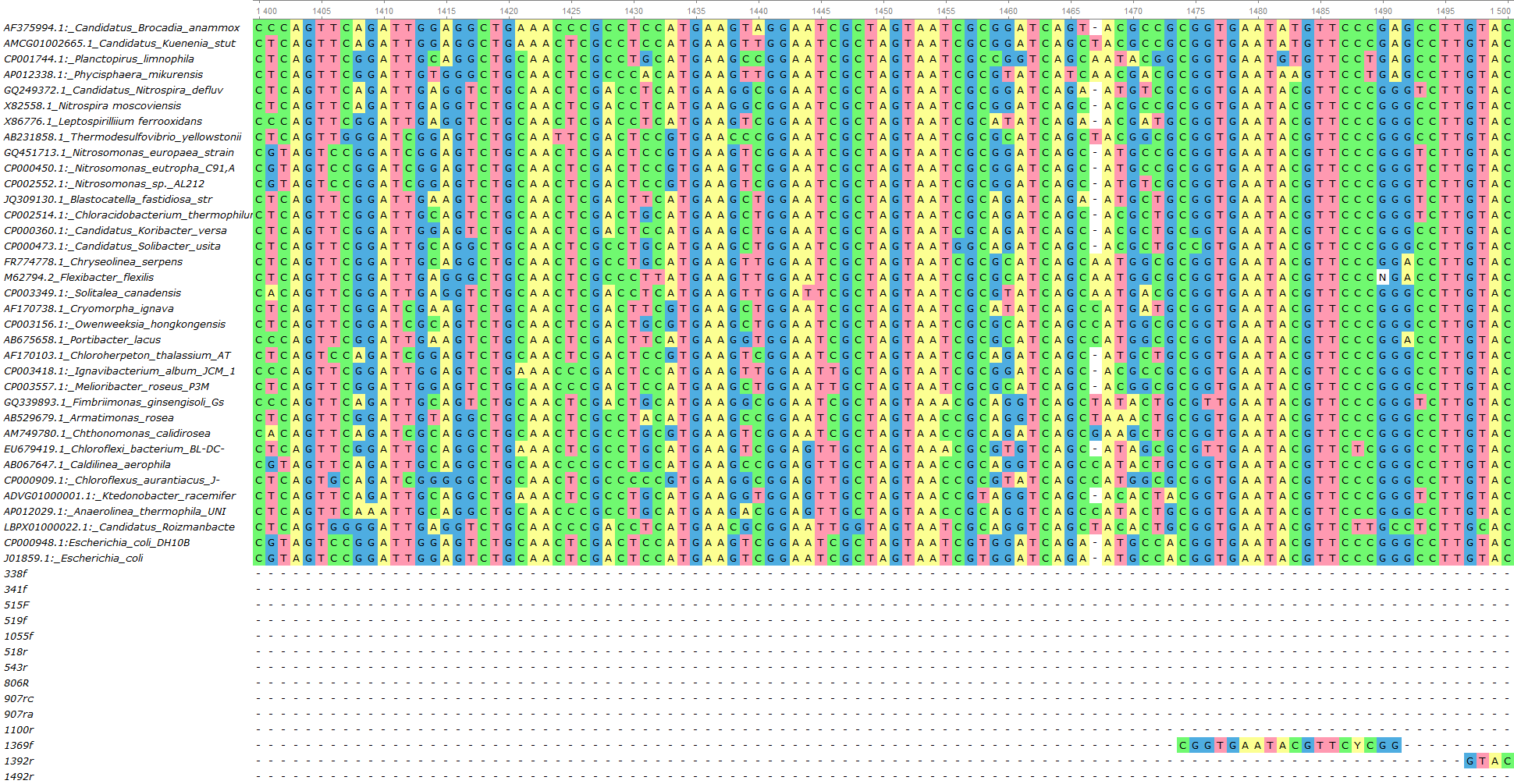

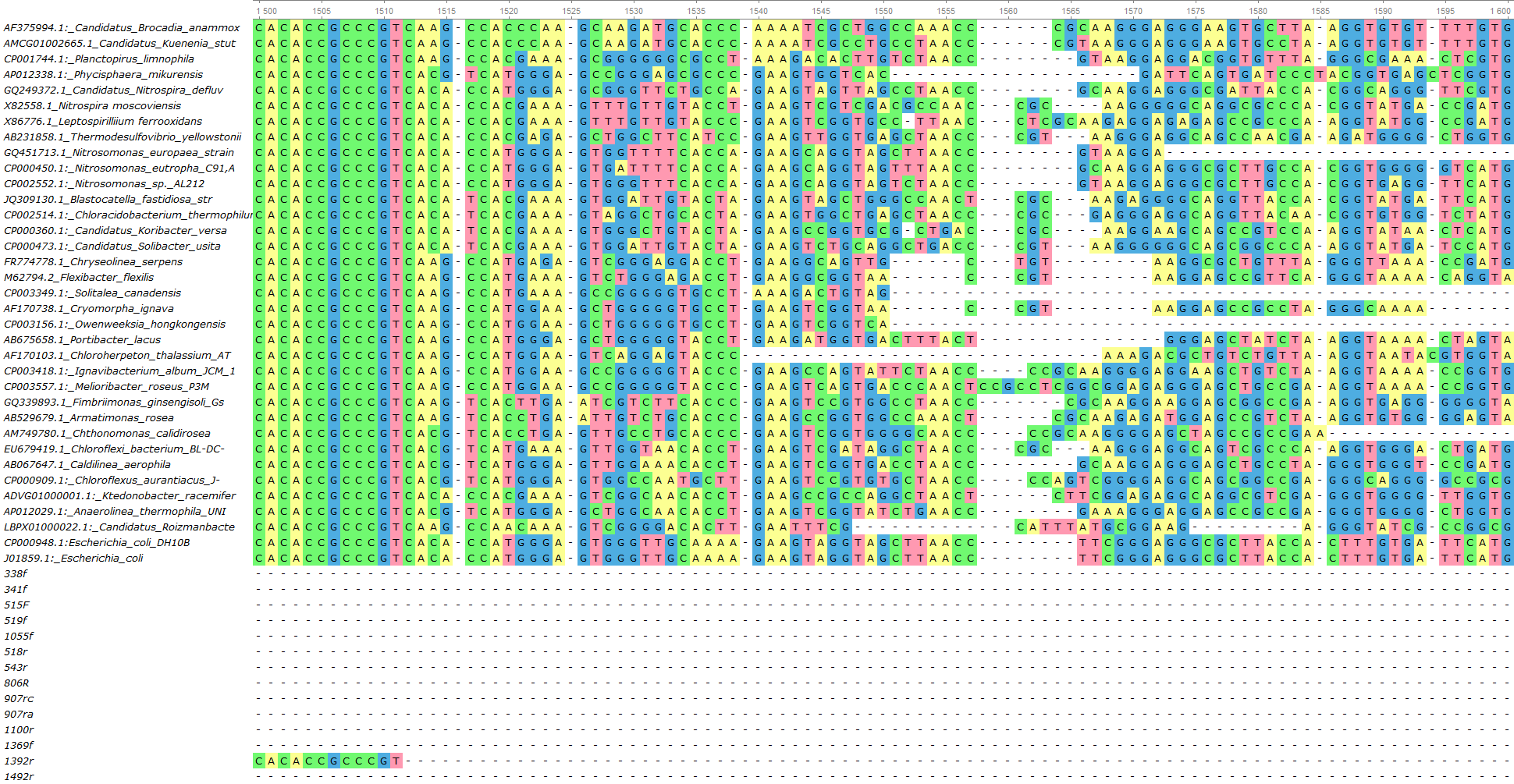

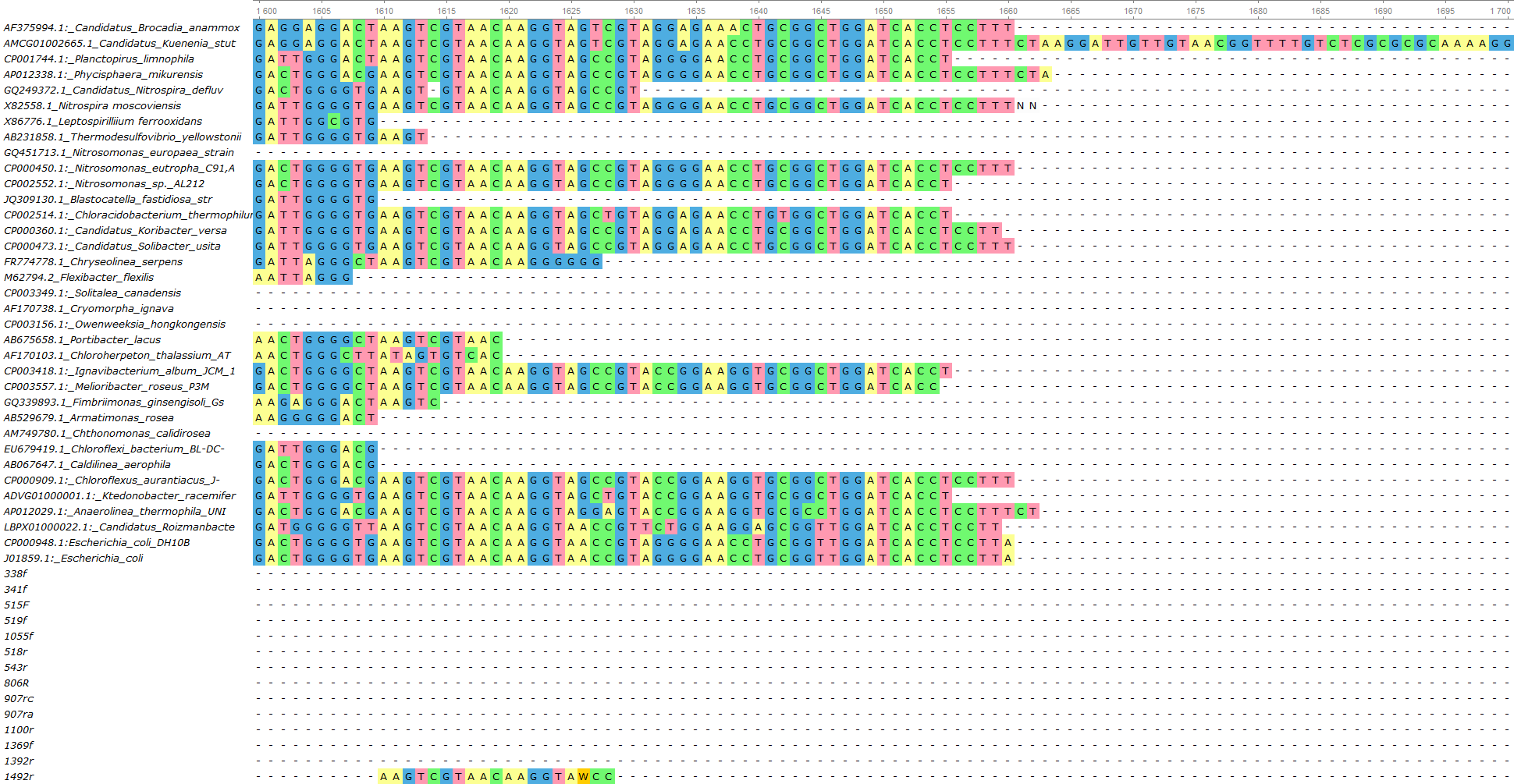


Supplementary Figure 2: Sequence alignment of the 16S rRNA gene from representatives of the PNA community according to Speth et al. (2017)^1^ and two E.coli sequences for verification of the primer position with all eubacterial primer sets found in literature assessment (Table 1). List of microbial members that were considered for the sequence alignment: (1) **Planctomycetes** – Candidatus Brocadia anammoxidans (AF375994.1), Phycisphaera mikurensis (AP012338.1), Planctopirus limnophila (CP001744.1), Candidatus Kuenenia stuttgartiensis (AMCG01002665.1); (2) **γ-Proteobacteria** – Nitrosomonas europaea strain ATCC 25978 (GQ451713.1), Nitrosomonas eutropha ATCC 19718 (CP000450.1); Nitrosomonas sp. AL212 (CP002552.1); (3) **Nitrospira** – Candidatus Nitrospira defluvii (GQ249372.1), Nitrospira moscoviensis (X82558.1), Thermodesulfovibrio yellowstonii (AB231858.1), Leptospirillum ferroxidans (X86776.1); (4) **Acidobacteria** – Blastocatella fastidiosa strain A2-16 (JQ309130.1), Chloracidobacterium thermophilum (CP002514.1), Candidatus Koribacter versatilis Ellin345 (CP000360.1), Candidatus Solibacter usitatus Ellin6076 (CP000473.1); (5) **Bacteriodetes** – Chryseolinea serpens (FR774778.1), Flexibacter flexilis (M62794.2), Solitalea canadensis (CP003349.1), Cryomorpha ignava (AF170738.1), Owenweeksia hongkongensis (CP003156.1), Portibacter lacus (AB675658.1); (6) **Chlorobi** – Chloroherpeton thalassium ATCC 35110 (AF170103.1), Ignavibacterium album JCM 16511 (CP003418.1), Melioribacter roseus P3M (CP003557.1); (7) **Armatimonadetes**– Fimbriimonas ginsengisoli Gsoil 348 (GQ339893.1), Armatimonas rosea (AB529679.1), Chthonomonas calidirosea (AM749780.1); (8) **Chloroflexi**– Chloroflexi bacterium BL-DC-9 (EU679419.1), Caldilinea aerophila (AB067647.1), Chloroflexus aurantiacus J-10-fl (CP000909.1), Ktedonobacter racemifer strain SOSP1-21 (ADVG01000001.1), Anaerolinea thermophila UNI-1 (AP012029.1); (9) **Parcubacterium** – Candidatus Roizmanbacteria bacterium GW2011_GWC2_35_12 UR63_C0022 (LBPX01000022.1); (10) **Escherichia coli** – Escherichia coli (J01859.1), Escherichia coli str. K12 substr. DH10B (CP000948.1)


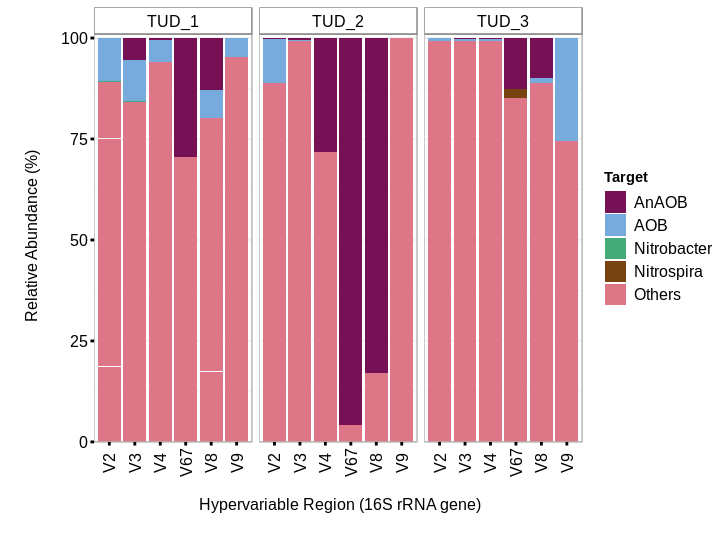


Supplementary Figure 3: Relative abundance of different microbial groups based on 16S rRNA gene amplicon sequencing, focusing on the microbial groups associated to PN/A systems, i.e. anaerobic ammonium oxidizing bacteria (AnAOB), ammonium oxidizing bacteria (AOB), nitrite oxidizing bacteria (Nitrobacter and Nitrospira) and others (rest of the microbial groups detected in the samples).


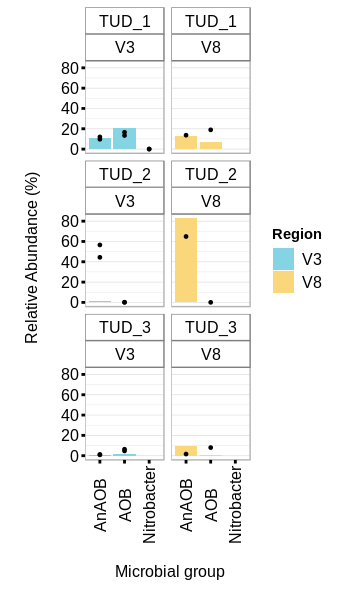


Supplementary Figure 4: comparison of relative abundance based on qPCR analysis and 16S rRNA amplicon sequencing. • represent the qPCR based relative abundance and the barplots represent the 16S rRNA based relative abundance. The represented microbial groups are associated to the PNA process, i.e. anaerobic ammonium oxidizing bacteria (AnAOB), ammonium oxidizing bacteria (AOB), nitrite oxidizing bacteria (Nitrobacter and Nitrospira).


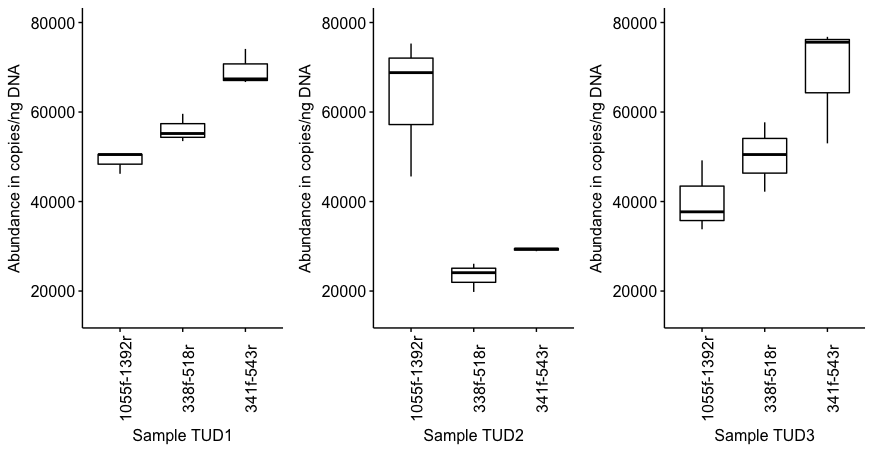


Supplementary Figure 5: qPCR based absolute abundance of samples TUD1, TUD2 and TUD 3 for eubacterial primer sets 1055f-1392r, 338f-518r and 341f-543r in copies/ng DNA; comparison of total eubacterial abundance measured using three different primer pairs (primer sets: (1) 1055f-1392r, (2) 338f-518r and (3) 341f-543r) for each sample.

Supplementary Table 1: Two-way ANOVA analysis to determine the effect of primer pair on the relative abundance based on16S rRNA gene amplicon sequencing analysis

| two-way ANOVA for 16S rRNA sequencing | | |
| --- | --- | --- |
|  | F-value | p-value |
|  | 109 | p<0.001 |

Supplementary Table 2: One-way ANOVA analysis to determine the effect of primer pairs on the measured abundance of EUB using qPCR analysis

| Sample | F-value | p-value |
| --- | --- | --- |
| TUD 1 | 29.08 | p<0.001 |
| TUD 2 | 16.39 | p<0.01 |
| TUD 3 | 6.074 | p<0.05 |

Supplementary Table 3: Overview of the evaluated studies conducted for the review the following keywords: “anammox and pcr” or “partial nitri* and pcr or nitritation” and “pcr or anaerobic ammoni* and pcr”

| **S.No.** | **List of Studies** |
| --- | --- |
| 1 | Hu, B. L., Zheng, P., Tang, C. J., Chen, J. W., van der Biezen, E., Zhang, L., ... & Kartal, B. (2010). Identification and quantification of anammox bacteria in eight nitrogen removal reactors. *Water research*, *44*(17), 5014-5020. |
| 2 | Suto, R., Ishimoto, C., Chikyu, M., Aihara, Y., Matsumoto, T., Uenishi, H., ... & Waki, M. (2017). Anammox biofilm in activated sludge swine wastewater treatment plants. *Chemosphere*, *167*, 300-307. |
| 3 | Ni, B. J., Hu, B. L., Fang, F., Xie, W. M., Kartal, B., Liu, X. W., ... & Yu, H. Q. (2010). Microbial and physicochemical characteristics of compact anaerobic ammonium-oxidizing granules in an upflow anaerobic sludge blanket reactor. *Applied and environmental microbiology*, *76*(8), 2652-2656. |
| 4 | An, P., Xu, X., Yang, F., Liu, L., & Liu, S. (2013). A pilot-scale study on nitrogen removal from dry-spun acrylic fiber wastewater using anammox process. *Chemical engineering journal*, *222*, 32-40. |
| 5 | Han, P., Huang, Y. T., Lin, J. G., & Gu, J. D. (2013). A comparison of two 16S rRNA gene-based PCR primer sets in unraveling anammox bacteria from different environmental samples. *Applied microbiology and biotechnology*, *97*(24), 10521-10529. |
| 6 | Bae, H., Park, K. S., Chung, Y. C., & Jung, J. Y. (2010). Distribution of anammox bacteria in domestic WWTPs and their enrichments evaluated by real-time quantitative PCR. *Process Biochemistry*, *45*(3), 323-334. |
| 7 | Park, H., Rosenthal, A., Jezek, R., Ramalingam, K., Fillos, J., & Chandran, K. (2010). Impact of inocula and growth mode on the molecular microbial ecology of anaerobic ammonia oxidation (anammox) bioreactor communities. *water research*, *44*(17), 5005-5013 |
| 8 | Ke, Y., Azari, M., Han, P., Görtz, I., Gu, J. D., & Denecke, M. (2015). Microbial community of nitrogen-converting bacteria in anammox granular sludge. *International Biodeterioration & Biodegradation*, *103*, 105-115. |
| 9 | Van der Star, W. R., Abma, W. R., Blommers, D., Mulder, J. W., Tokutomi, T., Strous, M., ... & van Loosdrecht, M. C. (2007). Startup of reactors for anoxic ammonium oxidation: experiences from the first full-scale anammox reactor in Rotterdam. *Water research*, *41*(18), 4149-4163. |
| 10 | Yang, Y. A. N. G., Zuo, J. E., Quan, Z. X., Lee, S., Shen, P., & Gu, X. (2006). Study on performance of granular ANAMMOX process and characterization of the microbial community in sludge. *Water science and technology*, *54*(8), 197-207. |
| 11 | Tsushima, I., Kindaichi, T., & Okabe, S. (2007). Quantification of anaerobic ammonium-oxidizing bacteria in enrichment cultures by real-time PCR. *Water Research*, *41*(4), 785-794. |
| 12 | Pathak, B. K., Kazama, F., Tanaka, Y., Mori, K., & Sumino, T. (2007). Quantification of anammox populations enriched in an immobilized microbial consortium with low levels of ammonium nitrogen and at low temperature. *Applied microbiology and biotechnology*, *76*(5), 1173-1179. |
| 13 | Tsushima, I., Ogasawara, Y., Shimokawa, M., Kindaichi, T., & Okabe, S. (2007). Development of a super high-rate Anammox reactor and in situ analysis of biofilm structure and function. *Water science and technology*, *55*(8-9), 9-17. |
| 14 | Li, X. R., Du, B., Fu, H. X., Wang, R. F., Shi, J. H., Wang, Y., ... & Quan, Z. X. (2009). The bacterial diversity in an anaerobic ammonium-oxidizing (anammox) reactor community. *Systematic and applied microbiology*, *32*(4), 278-289. |
| 15 | Ni, S. Q., Lee, P. H., Fessehaie, A., Gao, B. Y., & Sung, S. (2010). Enrichment and biofilm formation of Anammox bacteria in a non-woven membrane reactor. *Bioresource technology*, *101*(6), 1792-1799. |
| 16 | Yapsakli, K. (2010). Co-occurrence of nitrogen-converting organisms in full-scale treatment plants. *Journal of Environmental Science and Health Part A*, *45*(9), 1060-1070. |
| 17 | Terada, A., Lackner, S., Kristensen, K., & Smets, B. F. (2010). Inoculum effects on community composition and nitritation performance of autotrophic nitrifying biofilm reactors with counter‐diffusion geometry. *Environmental microbiology*, *12*(10), 2858-2872. |
| 18 | Ni, S. Q., Fessehaie, A., Lee, P. H., Gao, B. Y., Xu, X., & Sung, S. (2010). Interaction of anammox bacteria and inactive methanogenic granules under high nitrogen selective pressure. *Bioresource technology*, *101*(18), 6910-6915. |
| 19 | Zhang, T., Yan, Q. M., & Ye, L. (2010). Autotrophic biological nitrogen removal from saline wastewater under low DO. *Journal of chemical technology and biotechnology*, *85*(10), 1340-1345. |
| 20 | Ozdemir, B., Mertoglu, B., Yapsakli, K., Aliyazicioglu, C., Saatci, A., & Yenigun, O. (2011). Investigation of nitrogen converters in membrane bioreactor. *Journal of Environmental Science and Health, Part A*, *46*(5), 500-508. |
| 21 | Harhangi, H. R., Le Roy, M., van Alen, T., Hu, B. L., Groen, J., Kartal, B., ... & den Camp, H. J. O. (2012). Hydrazine synthase, a unique phylomarker with which to study the presence and biodiversity of anammox bacteria. *Applied and environmental microbiology*, *78*(3), 752-758. |
| 22 | Bürgmann, H., Jenni, S., Vazquez, F., & Udert, K. M. (2011). Regime shift and microbial dynamics in a sequencing batch reactor for nitritation/anammox treatment of urine. *Applied and environmental microbiology*, AEM-02986. |
| 23 | Ni, S. Q., Ni, J. Y., Hu, D. L., & Sung, S. (2012). Effect of organic matter on the performance of granular anammox process. *Bioresource technology*, *110*, 701-705. |
| 24 | Daverey, A., Su, S. H., Huang, Y. T., & Lin, J. G. (2012). Nitrogen removal from opto-electronic wastewater using the simultaneous partial nitrification, anaerobic ammonium oxidation and denitrification (SNAD) process in sequencing batch reactor. *Bioresource technology*, *113*, 225-231. |
| 25 | Liu, T., Li, D., Zeng, H., Li, X., Zeng, T., Chang, X., ... & Zhang, J. (2012). Biodiversity and quantification of functional bacteria in completely autotrophic nitrogen-removal over nitrite (CANON) process. *Bioresource Technology*, *118*, 399-406. |
| 26 | Chen, C. J., Huang, X. X., Lei, C. X., Zhu, W. J., Chen, Y. X., & Wu, W. X. (2012). Improving anammox start-up with bamboo charcoal. Chemosphere, 89(10), 1224-1229. |
| 27 | Daverey, A., Su, S. H., Huang, Y. T., Chen, S. S., Sung, S., & Lin, J. G. (2013). Partial nitrification and anammox process: a method for high strength optoelectronic industrial wastewater treatment. Water Research, 47(9), 2929-2937. |
| 28 | Ji, G., He, C., & Tan, Y. (2013). The spatial distribution of nitrogen removal functional genes in multimedia biofilters for sewage treatment. *Ecological engineering*, *55*, 35-42. |
| 29 | Huang, Y. T., Chen, S. S., Lee, P. H., & Bae, J. (2013). Microbial community and population dynamics of single-stage autotrophic nitrogen removal for dilute wastewater at the benchmark oxygen rate supply. *Bioresource technology*, *147*, 649-653. |
| 30 | Xie, B., Lv, Z., Hu, C., Yang, X., & Li, X. (2013). Nitrogen removal through different pathways in an aged refuse bioreactor treating mature landfill leachate. *Applied microbiology and biotechnology*, *97*(20), 9225-9234. |
| 31 | An, P., Xu, X., Yang, F., Liu, L., & Liu, S. (2013). A pilot-scale study on nitrogen removal from dry-spun acrylic fiber wastewater using anammox process. *Chemical engineering journal*, *222*, 32-40. |
| 32 | De Clippeleir, H., Vlaeminck, S. E., De Wilde, F., Daeninck, K., Mosquera, M., Boeckx, P., ... & Boon, N. (2013). One-stage partial nitritation/anammox at 15 C on pretreated sewage: feasibility demonstration at lab-scale. *Applied microbiology and biotechnology*, *97*(23), 10199-10210. |
| 33 | Persson, F., Sultana, R., Suarez, M., Hermansson, M., Plaza, E., & Wilén, B. M. (2014). Structure and composition of biofilm communities in a moving bed biofilm reactor for nitritation–anammox at low temperatures. *Bioresource technology*, *154*, 267-273. |
| 34 | Du, R., Peng, Y., Cao, S., Wu, C., Weng, D., Wang, S., & He, J. (2014). Advanced nitrogen removal with simultaneous Anammox and denitrification in sequencing batch reactor. *Bioresource technology*, *162*, 316-322. |
| 35 | Miao, L., Wang, K., Wang, S., Zhu, R., Li, B., Peng, Y., & Weng, D. (2014). Advanced nitrogen removal from landfill leachate using real-time controlled three-stage sequence batch reactor (SBR) system. *Bioresource technology*, *159*, 258-265. |
| 36 | Bi, Z., Qiao, S., Zhou, J., Tang, X., & Zhang, J. (2014). Fast start-up of Anammox process with appropriate ferrous iron concentration. *Bioresource technology*, *170*, 506-512. |
| 37 | Cydzik-Kwiatkowska, A., Rusanowska, P., Zielińska, M., Bernat, K., & Wojnowska-Baryła, I. (2014). Structure of nitrogen-converting communities induced by hydraulic retention time and COD/N ratio in constantly aerated granular sludge reactors treating digester supernatant. *Bioresource technology*, *154*, 162-170. |
| 38 | Pellicer‐Nàcher, C., Franck, S., Gülay, A., Ruscalleda, M., Terada, A., Al‐Soud, W. A., ... & Smets, B. F. (2014). Sequentially aerated membrane biofilm reactors for autotrophic nitrogen removal: microbial community composition and dynamics. *Microbial biotechnology*, *7*(1), 32-43. |
| 39 | Park, H., Sundar, S., Ma, Y., & Chandran, K. (2015). Differentiation in the microbial ecology and activity of suspended and attached bacteria in a nitritation‐anammox process. *Biotechnology and bioengineering*, *112*(2), 272-279. |
| 40 | Zeng, W., Li, B., Wang, X., Bai, X., & Peng, Y. (2014). Integration of denitrifying phosphorus removal via nitrite pathway, simultaneous nitritation–denitritation and anammox treating carbon-limited municipal sewage. *Bioresource technology*, *172*, 356-364. |
| 41 | Hendrickx, T. L., Kampman, C., Zeeman, G., Temmink, H., Hu, Z., Kartal, B., & Buisman, C. J. (2014). High specific activity for anammox bacteria enriched from activated sludge at 10 C. *Bioresource technology*, *163*, 214-221. |
| 42 | Li, H., Zhou, S., Ma, W., Huang, P., Huang, G., Qin, Y., ... & Ouyang, H. (2014). Long-term performance and microbial ecology of a two-stage PN–ANAMMOX process treating mature landfill leachate. *Bioresource technology*, *159*, 404-411. |
| 43 | Abbassi, R., Yadav, A. K., Huang, S., & Jaffé, P. R. (2014). Laboratory study of nitrification, denitrification and anammox processes in membrane bioreactors considering periodic aeration. Journal of environmental management, 142, 53-59. |
| 44 | Wang, H., Ji, G., Bai, X., & He, C. (2015). Assessing nitrogen transformation processes in a trickling filter under hydraulic loading rate constraints using nitrogen functional gene abundances. *Bioresource technology*, *177*, 217-223. |
| 45 | Ke, Y., Azari, M., Han, P., Görtz, I., Gu, J. D., & Denecke, M. (2015). Microbial community of nitrogen-converting bacteria in anammox granular sludge. *International Biodeterioration & Biodegradation*, *103*, 105-115. |
| 46 | Zekker, I., Rikmann, E., Tenno, T., Kroon, K., Seiman, A., Loorits, L., ... & Mandel, A. (2015). Start-up of low-temperature anammox in UASB from mesophilic yeast factory anaerobic tank inoculum. *Environmental technology*, *36*(2), 214-225. |
| 47 | Ren, L. F., Ni, S. Q., Liu, C., Liang, S., Zhang, B., Kong, Q., & Guo, N. (2015). Effect of zero-valent iron on the start-up performance of anaerobic ammonium oxidation (anammox) process. *Environmental Science and Pollution Research*, *22*(4), 2925-2934. |
| 48 | Wang, Y., Wang, Y., Wei, Y., & Chen, M. (2015). In-situ restoring nitrogen removal for the combined partial nitritation-anammox process deteriorated by nitrate build-up. *Biochemical engineering journal*, *98*, 127-136. |
| 49 | Marie, P. S., Pümpel, T., Markt, R., Murthy, S., Bott, C., & Wett, B. (2015). Comparative evaluation of multiple methods to quantify and characterise granular anammox biomass. *water research*, *68*, 194-205. |
| 50 | Zhang, L., Zhang, S., Peng, Y., Han, X., & Gan, Y. (2015). Nitrogen removal performance and microbial distribution in pilot-and full-scale integrated fixed-biofilm activated sludge reactors based on nitritation-anammox process. *Bioresource technology*, *196*, 448-453. |
| 51 | Cydzik-Kwiatkowska, A., & Wojnowska-Baryła, I. (2015). Nitrogen-converting communities in aerobic granules at different hydraulic retention times (HRTs) and operational modes. *World Journal of Microbiology and Biotechnology*, *31*(1), 75-83. |
| 52 | Guimarães, L. B., Mezzari, M. P., Daudt, G. C., & da Costa, R. H. (2017). Microbial pathways of nitrogen removal in aerobic granular sludge treating domestic wastewater. *Journal of Chemical Technology & Biotechnology*, *92*(7), 1756-1765. |
| 53 | Xiao, P., Lu, P., Zhang, D., Han, X., & Yang, Q. (2015). Effect of trace hydrazine addition on the functional bacterial community of a sequencing batch reactor performing completely autotrophic nitrogen removal over nitrite. *Bioresource technology*, *175*, 216-223. |
| 54 | Ma, Y., Sundar, S., Park, H., & Chandran, K. (2015). The effect of inorganic carbon on microbial interactions in a biofilm nitritation–anammox process. *Water research*, *70*, 246-254. |
| 55 | Daverey, A., Chen, Y. C., Dutta, K., Huang, Y. T., & Lin, J. G. (2015). Start-up of simultaneous partial nitrification, anammox and denitrification (SNAD) process in sequencing batch biofilm reactor using novel biomass carriers. Bioresource technology, 190, 480-486. |
| 56 | Chen, H., Hu, H. Y., Chen, Q. Q., Shi, M. L., & Jin, R. C. (2016). Successful start-up of the anammox process: influence of the seeding strategy on performance and granule properties. *Bioresource technology*, *211*, 594-602. |
| 57 | Yin, X., Qiao, S., Zhou, J., & Tang, X. (2016). Fast start-up of the anammox process with addition of reduced graphene oxides. *Chemical Engineering Journal*, *283*, 160-166. |
| 58 | Ren, L. F., Lv, L., Zhang, J., Gao, B., Ni, S. Q., Yang, N., ... & Liu, X. (2016). Novel zero-valent iron-assembled reactor for strengthening anammox performance under low temperature. *Applied microbiology and biotechnology*, *100*(20), 8711-8720. |
| 59 | Wang, Z., Peng, Y., Miao, L., Cao, T., Zhang, F., Wang, S., & Han, J. (2016). Continuous-flow combined process of nitritation and ANAMMOX for treatment of landfill leachate. *Bioresource technology*, *214*, 514-519. |
| 60 | Wang, X., & Gao, D. (2016). In-situ restoration of one-stage partial nitritation-anammox process deteriorated by nitrate build-up via elevated substrate levels. *Scientific reports*, *6*, 37500. |
| 61 | Wang, Z. B., Ni, S. Q., Zhang, J., Zhu, T., Ma, Y. G., Liu, X. L. & Miao, M. S. (2016). Gene expression and biomarker discovery of anammox bacteria in different reactors. *Biochemical engineering journal*, *115*, 108-114. |
| 62 | Miao, L., Wang, S., Cao, T., Peng, Y., Zhang, M., & Liu, Z. (2016). Advanced nitrogen removal from landfill leachate via Anammox system based on Sequencing Biofilm Batch Reactor (SBBR): Effective protection of biofilm. *Bioresource technology*, *220*, 8-16. |
| 63 | Wu, S., Bhattacharjee, A. S., Weissbrodt, D. G., Morgenroth, E., & Goel, R. (2016). Effect of short term external perturbations on bacterial ecology and activities in a partial nitritation and anammox reactor. *Bioresource technology*, *219*, 527-535. |
| 64 | Reeve, P. J., Mouilleron, I., Chuang, H. P., Thwaites, B., Hyde, K., Dinesh, N., & van den Akker, B. (2016). Effect of feed starvation on side-stream anammox activity and key microbial populations. *Journal of environmental management*, *171*, 121-127. |
| 65 | Wang, B., Peng, Y., Guo, Y., Yuan, Y., Zhao, M., & Wang, S. (2016). Impact of partial nitritation degree and C/N ratio on simultaneous Sludge Fermentation, Denitrification and Anammox process. *Bioresource technology*, *219*, 411-419. |
| 66 | Miao, Y., Zhang, L., Yang, Y., Peng, Y., Li, B., Wang, S., & Zhang, Q. (2016). Start-up of single-stage partial nitrification-anammox process treating low-strength swage and its restoration from nitrate accumulation. *Bioresource technology*, *218*, 771-779. |
| 67 | Leal, C. D., Pereira, A. D., Nunes, F. T., Ferreira, L. O., Coelho, A. C. C., Bicalho, S. K., ... & de Araújo, J. C. (2016). Anammox for nitrogen removal from anaerobically pre-treated municipal wastewater: effect of COD/N ratios on process performance and bacterial community structure. *Bioresource technology*, *211*, 257-266. |
| 68 | Wang, H., Ji, G., & Bai, X. (2016). Distribution patterns of nitrogen micro-cycle functional genes and their quantitative coupling relationships with nitrogen transformation rates in a biotrickling filter. *Bioresource technology*, *209*, 100-107. |
| 69 | Liu, T., Li, D., Zhang, J., Lv, Y., & Quan, X. (2016). Effect of temperature on functional bacterial abundance and community structure in CANON process. *Biochemical engineering journal*, *105*, 306-313. |
| 70 | Regmi, P., Holgate, B., Miller, M. W., Park, H., Chandran, K., Wett, B. & Bott, C. B. (2016). Nitrogen polishing in a fully anoxic anammox MBBR treating mainstream nitritation–denitritation effluent. *Biotechnology and bioengineering*, *113*(3), 635-642. |

Supplementary Table 4: Real-time Primer sequences with reaction conditions used in this study

| **Primer Pair** | **Annealing Temperature (°C)** | **Primer name/Sequence (5’-3’)** | **References** |
| --- | --- | --- | --- |
| 1055f-1392r | 55 | 1055f (ATGGCTGTCGTCAGCT)  1392r (ACGGGCGGTGTGTAC) | 2 |
| 341f-543r | 62 | 341f (CCTACGGGAGGCAGCAG)  543r (TTACCGCGGCTGCTGGCAC) | 3 |
| 338f-518r | 64 | 338f (ACTCCTACGGGGAGGCAGCA)  518r (ATTACCGCGGCTGCTGG) | 4 |
| amoA1f-amoA2r | 55 | amoA1f (GGGGTTTCTACTGGTGGT)  amoA2r CCCCTCKGSAAAGCCTTCTTC) | 5 |
| Amx809f-Amx1066r | 65 | Amx809f (GCCGTAAACGATGGGCACT)  Amx1066r (AACGTCTCACGACACGAGCTG) | 6 |
| NSR1113f-NSR1265r | 68 | NSR1113f (CCTGCTTTCAGTTGCTACCG)  NSR1265r (GTTTGCAGCGCTTTGTACCG) | 7 |
| Nitro1198f-Nitro1423r | 68 | Nitro1198f (ACCCCTAGCAAATCTCAAAAACCG)  Nitro1423r (CTTCACCCCAGTCGCTGACC) | 8 |

1. References

1. Speth, D.R., in 't Zandt, M.H., Guerrero-Cruz, S., Dutilh, B.E. & Jetten, M.S.M. Genome-based microbial ecology of anammox granules in a full-scale wastewater treatment system. *Nat. Commun.* **7**, 10 (2016).

2. Ferris, M., Muyzer, G. & Ward, D. Denaturing gradient gel electrophoresis profiles of 16S rRNA-defined populations inhabiting a hot spring microbial mat community. *Appl. Environ. Microbiol.* **62**, 340-346 (1996).

3. Koike, S. et al. Monitoring and source tracking of tetracycline resistance genes in lagoons and groundwater adjacent to swine production facilities over a 3-year period. *Appl. Environ. Microbiol.* **73**, 4813-4823 (2007).

4. Muyzer, G., de Waal, E.C. & Uitterlinden, A.G. Profiling of complex microbial populations by denaturing gradient gel electrophoresis analysis of polymerase chain reaction-amplified genes coding for 16S rRNA. *Appl Environ Microbiol* **59**, 695-700 (1993).

5. Rotthauwe, J.H., Witzel, K.P. & Liesack, W. The ammonia monooxygenase structural gene amoA as a functional marker: Molecular fine-scale analysis of natural ammonia-oxidizing populations. *Appl. Environ. Microbiol.* **63**, 4704-4712 (1997).

6. Tsushima, I., Kindaichi, T. & Okabe, S. Quantification of anaerobic ammonium-oxidizing bacteria in enrichment cultures by real-time PCR. *Water Res.* **41**, 785-794 (2007).

7. Kindaichi, T., Kawano, Y., Ito, T., Satoh, H. & Okabe, S. Population dynamics and in situ kinetics of nitrifying bacteria in autotrophic nitrifying biofilms as determined by real-time quantitative PCR. *Biotechnol Bioeng* **94**, 1111-1121 (2006).

8. Knapp, C.W. & Graham, D.W. Nitrite-oxidizing bacteria guild ecology associated with nitrification failure in a continuous-flow reactor. *FEMS Microbiol. Ecol.* **62**, 195-201 (2007).
